# Extended-spectrum Beta-lactamase and AmpC beta-lactamases producing gram negative bacilli isolated from clinical specimens at International Clinical Laboratories, Addis Ababa, Ethiopia

**DOI:** 10.1371/journal.pone.0241984

**Published:** 2020-11-12

**Authors:** Saba Gebremichael Tekele, Dejenie Shiferaw Teklu, Kassu Desta Tullu, Samuel Kinde Birru, Melese Hailu Legese

**Affiliations:** 1 Department of Medical Laboratory Sciences, College of Health Sciences, Wollo University, Dessie, Ethiopia; 2 Department of Bacteriology and Mycology, Ethiopian Public Health Institute, Addis Ababa, Ethiopia; 3 Department of Medical Laboratory Sciences, College of Health Sciences, Addis Ababa University, Addis Ababa, Ethiopia; University of Nicolaus Copernicus in Torun, POLAND

## Abstract

**Background:**

Extended spectrum Beta-lactamases (ESBLs) and AmpC beta-lactamases (AmpC) are the common enzymes produced by gram negative bacilli, which are their main mechanisms of resistance to all generations of cephalosporins. Hence, this study aimed to determine the magnitude of ESBLs and AmpC producing gram negative bacilli (GNB) isolated from clinical specimens at International clinical Laboratories in Addis Ababa, Ethiopia.

**Methods:**

A cross sectional study was conducted from January to May 2018. From different clinical specimens, 338 GNB were isolated and characterized. Bacterial species identification, antimicrobial susceptibility testing and screening for ESBLs and AmpC production were performed using Phoenix automated system (BD phoenix_100_). ESBLs production was confirmed using a combination disc method. All Cefoxitin resistant and confirmed ESBLs producing GNB were confirmed for AmpC beta-lactamases production by AmpC confirmatory Neo-Sensitabs discs (ROSCO tablet). Data were analyzed using SPSS version 20 software.

**Results:**

*E*. *coli* 66.0% (224/338) followed by *K*. *pneumoniae* 12.1% (41/338) were GNB most frequently isolated. The overall magnitude of ESBLs producing GNB was 38.8% (131/338) and the extent of AmpC beta-lactamase producing GNB was 2.4% (8/338). Majority of ESBLs and AmpC beta-lactamases producing GNB were isolated from urine specimens 47.5% (116/338). Ampicillin (75.4%), amoxicillin with clavulanic acid (64.0%) and sulfamethoxazole-trimethoprim (55.6%) were most the antibiotics to which resistance was most commonly found. The multidrug resistance (MDR) level of GNB was 74.0% (250/338). Of ESBLs and AmpC beta-lactamases producing GNB, 99.3% were MDR (p < 0.05).

**Conclusion:**

The high magnitude of ESBLs and AmpC beta-lactamases producing GNB calls the needs of strong intervention to minimize further occurrence and spread of such GNB. More importantly, the MDR level was high which suggests continuous monitoring & reviewing of antimicrobial policy in hospitals and the country at large.

## Background

Gram negative bacteria such as *Escherichia coli*, *Klebsiella pneumoniae*, *Pseudomonas aeruginosa* and *Acinetobacter baumannii* are among the most important pathogens causing nosocomial and community acquired bacterial infections in humans. These bacteria are becoming resistant to many available antimicrobial agents which are a major public health threat nowadays [1, 2].

Beta-lactam antibiotics are the most commonly used antibiotics to treat infections caused by such multidrug resistant gram negative bacilli. However, resistance to beta-lactam antibiotics due to the production of beta-lactamases is becoming a worldwide threat. Antibiotic resistance can be developed when bacterial genes mutate continuously in response to overuse or misuse of beta-lactam antibiotics [3–5]. Although there are more than 1000 beta-lactamase enzymes, ESBLs and AmpC beta-lactamases are the most common enzymes produced by GNB [6, 7].

ESBLs have the ability to hydrolyze most beta-lactam antibiotics such as cephalosporins, penicillins, aztreonam and related oxyimino beta-lactams except cephamycins (cefoxitin) and carbapenems which can only be inhibited by beta-lactamase inhibitors such as clavulanic acid [8]. ESBLs are most often produced by *Klebsiella spp*. and *E*. *coli al*though *Enterobacter spp*, *Citrobacter spp*, *Morganella spp*, *Serratia spp*, *Pseudomonas* spp and other gram negative bacilli are also are ESBLs producers [9]. In addition, ESBLs producing isolates exhibit co-resistance to other classes of antibiotics such as aminoglycosides, fluoroquinolones and sulfonamides [10].

Like ESBLs, the class C beta-lactamases (AmpC) are clinically significant because GNB which confer AmpC are also resistant to penicillin, cephalosporin, cephamycin and monobactam groups. In contrast to ESBL, AmpC beta-lactamase activity is not affected by ESBLs inhibitors [11]. AmpC beta-lactamase can be chromosomal or plasmid-mediated. Chromosomally mediated AmpC beta-lactamase are found in *Serratia*, *Pseudomonas*, *Acinetobacter*, *Citrobacter* and *Enterobacter spp*. Plasmid mediated AmpC beta-lactamase are commonly seen in *Enterobacteriaceae* including *E*. *coli*, *K*. *pneumoniae*, *Salmonella spp*., *Citrobacter freundii*, and *Proteus mirabilis* [12, 13].

An important additional issue is that, Amp-C producing GNB acts as a hidden reservoir for ESBLs. Therefore, co-existence of these enzymes complicates the treatment of GNB as Amp-C beta-lactams may mask the recognition of ESBLs [2]. The production of ESBLs and AmpC beta-lactamases can be detected using conventional and automated techniques but automated systems such as Phoenix 100, Vitek-2, and MicroScan WalkAway 96 Plus are used for rapid identification of bacterial species and antimicrobial susceptibility testing in microbiology laboratories [14, 15].

In general, GNB that produce ESBLs and AmpC enzymes cause treatment difficulties and treatment failure, including morbidity and mortality. Therefore, detecting and determining the magnitude of ESBLs and AmpC is crucial for effective treatment and for prevention and control of these resistant bacteria. Additionally, adequate knowledge about the magnitude of ESBLs and AmpC is important to limit the spread and further prevalence of ESBLs and AmpC production in GNB. Hence, the present study aimed to determine the magnitude of ESBLs and AmpC producing GNB isolated from clinical specimens at International Clinical Laboratories in Addis Ababa, Ethiopia.

## Methods

A cross sectional study was conducted from January 2018 to May 2018 at International Clinical Laboratories (ICL) in Addis Ababa, Ethiopia. ICL was established in 2004 and is one of the main clinical laboratories in Ethiopia which gives numerous services including microbiological analysis. Its microbiology department receives clinical specimens for microbiological analysis from different governmental and private hospitals in Addis Ababa. It is equipped with several automated machines including the Phoenix automated system that is used for bacterial identification and antimicrobial susceptibility testing. During the study period, a total of 338 GNB isolates were isolated from different clinical samples and included in this study for ESBLs and AmpC characterization and antimicrobial susceptibility testing.

The sample size was calculated based on single-population proportion using a previous study done in Ethiopia [16]. The socio-demographic data of patients was documented using the data collection sheet from the request form. The GNB isolates, the types of beta-lactamases and the antibiotics susceptibility pattern of the isolate were also recorded using a separate data collection sheet.

### Isolation and identification of gram negative bacilli

The GNB isolates were obtained from various clinical specimens such as urine, pus, body fluids, sputum, stool, ear and eye discharges. The specimens were inoculated in to 5% sheep blood agar, XLD agar and MacConkey agar plates (all media were Oxoid Ltd, UK) and incubated at 37°C for 18–24 hours. Each bacterium was characterized by colony appearance, size, consistency, shape, ability to ferment lactose and gram stain. All bacterial species were characterized and identified to species level using the Phoenix system (BD Diagnostic Systems, Oxford, UK) by adjusting the turbidity of bacterial suspension to 0.5 McFarland standards inoculum density. Bacteria suspension was prepared from pure colonies grown on primary isolation media and inoculated to the appropriate phoenix panel [17].

### Antimicrobial susceptibility testing

Antimicrobial susceptibility testing for 17 antimicrobials namely ceftazidime, cefotaxime, cefuroxime, ciprofloxacin, ceftriaxone, cefepime, amoxicillin with clavulanic acid, amikacin, aztreonam, ertapenem, cefoxitin, gentamicin, imipenem, meropenem, ampicillin, sulfametoxazole-trimethoprim and piperacillin/ tazobactam was performed using Phoenix AST panel (AST-N94) that applies the microdilution method. The Phoenix machine also has a screening test for ESBL and AmpC beta-lactamase production among GNB [17].

### Screening for ESBLs production

According to the CLSI guidelines, isolates that showed an MIC ≥2μg/ml for ceftazidime and/or for cefotaxime were considered as suspected for ESBL producer [18].

### Confirmation of ESBLs production

A disc of ceftazidime (30 μg) and cefotaxime (30 μg) alone and in combination with clavulanic acid (30 μg/10 μg) and cefotaxime (30 μg) + clavulanic acid (30 μg/10 μg) were placed at a distance of 25 mm apart on a Muller Hinton agar plate inoculated with bacterial suspension of 0.5 McFarland turbidity standards and incubated overnight (18–24 hrs.) at 37°C. An increase in the inhibition zone diameter of ≥5 mm for a combination disc versus ceftazidime or cefotaxime disk alone was confirmed as ESBL producing bacilli according to CLSI (2018) guidelines [18].

### Screening of AmpC production

Guided by EUCAST, isolates showing reduced susceptibility to cefoxitin (MIC >8 μg/ml) were identified as a potential AmpC beta-lactamase producer. In addition, all ESBLs suspected isolates were screened for confirmation [19].

### Confirmation of AmpC production

All cefoxitin non-susceptible isolates and ESBLs suspected isolates were checked for the presence of AmpC beta-lactamase using disc diffusion tablets Neo-Sensitabs (ROSCO, Taastrupgaardsvej 30, DK-2630 Taastrup, Denmark) one tablet with cefotaxime, one with ceftazidime and two tablets of cephalosporins combined with cloxacillin (AmpC inhibitor). An increase in the inhibition zone diameter of ≥5 mm for a combination disc versus ceftazidime or cefotaxime disk alone was confirmed as AmpC beta-lactamase producing GNB [20].

### Data quality assurance

After preparation, culture media was checked for sterility by incubating 5% of them overnight and observing them for the presence of any bacterial growth. Quality control for the Phoenix machine and prepared media was performed using standard ATCC control strains.

For ESBL testing, *K*. *pneumoniae* ATCC 700603 was used as a (positive control) and *E*. *coli* ATCC 25922 was used as a (negative control) strains. *Enterobacter cloacae* (ATCC BAA 1143) and *E*. *coli* (ATCC 25922) were used as positive and negative QC strains for AmpC beta-lactamase producing GNB respectively. Before applying the 20% glycerol TSB for storage, it was QC tested for growth of *E*. *coli* ATCC 25922 standard strains. Data was re-assessed for its completeness, adequate recoding on the worksheet and in the SPSS software after entry.

### Data analysis and interpretation

Data was analyzed using SPSS version 20 software. Descriptive statistics were summarized in tables and graphs. Proportions and actual number of AmpC and ESBLs producing GNB isolates were used to describe frequency of categorical variables. Continuous variables were described by mean and standard deviation.

### Ethical considerations

The study was conducted after obtaining ethical clearance from the research and ethical review committee of the department of Medical Laboratory Sciences, School of Allied Health Sciences, College of Health Sciences; Addis Ababa University (DRERC/335/18/MLS). Additionally, an official permission letter was obtained from the study site.

## Results

### Socio-demographic characteristics

During the study period, a total of 338 GNB isolates were isolated from various clinical specimens sent to ICL by government hospitals (n = 151) and private hospitals (n = 187). The majority of the GNB were isolated from urine 72.2% (244/338) followed by pus 18.6% (63/338) (**Table 1**).

**Table 1:** Distribution of gram negative bacilli isolate against demographic characteristics and specimen types.

Gram negative bacilli n (%)												
Variables (Number)		*E*.*coli*	*K*.*pneumoniae*	*K*.*oxytoca*	*K*.*ozonea*	*Pseudomonas* spp.	*Enterobacter* spp.	*Citrobacter* spp.	*Acinetobacter* spp.	*P*.*mirabilis*	*Shigella* spp.	Other Isolate
Sex	Male(141)	89(63.1)	13(9.2)	1(0.7)	0(0.00)	5(3.5)	8(5.7)	7(5.0)	5(3.5)	1(0.7)	2(1.4)	10(7.1)
	Female(197)	135(68.5)	28(14.2)	3(1.5)	1(0.5)	12(6.1)	3(1.5)	3(1.5)	4(2.0)	4(2.0)	0(0.0)	5(2.5)
Age group	≤15(35)	18(51.4)	4(11.4)	3(8.6)	0(0.0)	2(5.7)	0(0.0)	1(2.9)	2(5.7)	3(8.6)	0(0.0)	3(8.6)
	16-<32(73)	52(71.2)	6(8.2)	1(1.4)	1(1.4)	2(2.7)	4(5.5)	0(0.0)	3(4.1)	2(2.7)	1(1.4)	1(1.4)
	32-<46(74)	49(66.2)	9(12.2)	0(0.0)	0(0.0)	3(4.1)	4(5.4)	4(5.4)	2(2.7)	0(0.0)	1(1.4)	2(2.7)
	46-<61(70)	43(61.4)	10(14.3)	0(0.0)	0(0.0)	7(10.0)	1(1.4)	2(2.9)	1(1.4)	0(0.0)	0(0.0)	6(8.6)
	≥61(86)	62(72.1)	12(14.0)	0(0.0)	0(0.0)	3(3.5)	2(2.3)	3(3.5)	1(1.2)	0(0.0)	0(0.0)	3(3.5)
Types of Specimen	Urine(244)	189(77.5)	23(9.4)	1(0.4)	0(0.0)	4(1.6)	5(2.0)	8(3.3)	4(1.6)	2(0.8)	0(0.0)	8(3.3)
	Pus (63)	24(38.1)	11(17.5)	2(3.2)	0(0.0)	7(11.1)	6(9.5)	2(3.2)	4(6.3)	1(1.6)	0(0.0)	6(9.5)
	Body fluid(10)	7(70.0)	2(20.0)	0(0.0)	0(0.0)	0(0.0)	0(0.0)	0(0.0)	0(0.0)	0(0.0)	0(0.0)	1(10.0)
	Discharge(13)	1(7.7)	3(23.1)	1(7.7)	1(100)	5(38.5)	0(0.0)	0(0.0)	1(7.7)	2(15.4)	0(0.0)	0(0.0)
	Sputum(6)	3(50.0)	2(33.3)	0(0.0)	0(0.0)	1(16.7)	0(0.0)	0(0.0)	0(0.0)	0(0.0)	0(0.0)	0(0.0)
	Stool(2)	0(0.0)	0(0.0)	0(0.0)	0(0.0)	0(0.0)	0(0.0)	0(0.0)	0(0.0)	0(0.0)	2(100)	0(0.0)
	**Total(N = 338)**	**224(66.3)**	**41(12.1)**	**4(1.2)**	**1(0.3)**	**17(5.0)**	**11(3.3)**	**10(3.0)**	**9(2.7)**	**5(1.5)**	**2(0.6)**	**15(4.4)**

Note: *other isolates are *Salmonella* spp., *Providencia* spp., *M*. *morganii*, *Serratia* spp.

Among the total isolates, 58.3% (n = 197/338) were isolated from female patients and 41.7% (n = 141/338) were collected from male patients. *E*. *coli* (68.5%) and *K*. *pneumoniae* (14.2%) were the most frequently isolated bacteria from females. The majority of isolates 86 (25.4%) were obtained from patients above 61 years of age. The mean age of patients was 43.9 years and standard deviation 21.8 years (Table 1).

### Frequency of gram negative bacilli isolates

Among the total 338 GNB isolates, *E*. *coli* with 66.3% (224/338) was the most frequent isolates followed by *K*. *pneumoniae* with 12.1% (41/338). From the 224 *E*. *coli* isolates, 84.4% (189/224) were isolated from urine and 10.7% (24/224) were isolated from pus specimens. Among the non-fermenting GNB, *Pseudomonas* spp. were obtained from pus in 41.2% (7/17), from discharge in 29.4% (5/17) and from urine in 23.5% (4/17) **(Table 1).**

### Antibiotics resistance pattern of gram negative bacilli isolates

For all isolates, drug susceptibility testing was performed using the automated Phoenix system which has a broth microdilution method that gives MIC. Highest resistance level was recorded to ampicillin (75.4%), amoxicillin with clavulanic acid (64.0%), sulfamethoxazole-trimethoprim (55.6%), cefuroxime (48.2%) and cefotaxime (47.0%). *E*. *coli* showed the highest resistance to ampicillin (77.2%) followed by amoxicillin with clavulanic acid (67.9%). In *K*. *pneumoniae* isolates the highest level of resistance was observed against ampicillin (100%), amoxicillin with clavulanic acid (73.2%), sulfamethoxazole-trimethoprim (70.7%), cefuroxime (65.9%) and aztreonam (63.4%). These isolates showed a lower resistance level to meropenem(9.8%), ertapenem (9.8%) and imipenem (12.2%).No resistance was observed to amikacin (0.0%) **(Table 2)**.

**Table 2:** Antimicrobial resistance pattern of gram negative bacilli.

Isolates (N)	CRO	CAZ	FEP	CTX	CXM	FOX	MER	IMP	ETP	SXT	CIP	GM	AMP	AMC	AN	ATM	TZP
***E*.*coli*(n = 224)**	110 (49.1)	108 (48.2)	110 (49.1)	114 (50.9)	115 (51.3)	20 (8.9)	2 (0.9)	1 (0.4)	5 (2.2)	135 (60.3)	92 (41.1)	43 (19.2)	173 (77.2)	152 (67.9)	2 (0.9)	114 (50.9)	15 (6.7)
***K*.*pneumoniae* (n = 41)**	26 (63.4)	25 (61.0)	25 (61.0)	26 (63.4)	27 (65.9)	7 (17.1)	4 (9.8)	5 (12.2)	4 (9.8)	29 (70.7)	22 (53.6)	12 (28.6)	41 (100.0)	30 (73.2)	0 (0.0)	26 (63.4)	8 (19.5)
***K*.*oxytoca(n* = 3*)***	0 (0.0)	0 (0.0)	0 (0.0)	0 (0.0)	0 (0.0)	0 (0.0)	0 (0.0)	0 (0.0)	0 (0.0)	0 (0.0)	0 (0.0)	0 (0.0)	2 (50.0)	0 (0.0)	0 (0.0)	0 (0.0)	0 (0.0)
***K*.** ozaenae **(n = 1)**	0 (0.0)	0 (0.0)	0 (0.0)	0 (0.0)	0 (0.0)	0 (0.0)	0 (0.0)	0 (0.0)	0 (0.0)	0 (0.0)	0 (0.0)	0 (0.0)	0 (0.0)	0 (0.0)	0 (0.0)	0 (0.0)	0 (0.0)
***Pseudomonas* spps.(n = 17)**	NA	4 (23.5)	4 (23.5)	NA	NA	NA	1 (5.9)	0 (0.0)	NA	NA	3 (17.6)	2 (11.8)	NA	NA	2 (11.8)	4 (23.5)	3 (17.6)
***Enterobacter Spp*.(n = 11)**	8 (72.7)	5 (45.5)	7 (63.6)	8 (72.7)	8 (72.7)	9 (81.8)	0 (0.0)	0 (0.0)	1 (9.0)	8 (72.7)	5 (45.5)	6 (54.5)	11 (100.0)	11 (100)	0 (0.0)	8 (72.7)	2 (18.2)
***Citrobacter* spps.(n = 10)**	2 (20.0)	2 (20.0)	1 (10.0	5 (50.0)	4 (40.0)	4 (40.0)	0 (0.0)	2 (20.0)	0 (0.0)	6 (60.0)	5 (50.0)	2 (20.0)	10 (100.0)	9 (90.0)	0 (0.0)	5 (50.0)	1 (10.0)
***Acinetobacter* spps.(n = 9)**	NA	2 (22.2)	3 (33.3)	NA	NA	NA	0 (0.0)	0 (0.0)	NA	NA	2 (22.2)	2 (22.2)	NA	NA	1 (11.1)	NA	2 (22.2)
***P*.*mirabilis* (n = 5)**	1 (20.0)	0 (0.0)	1 (20.0)	1 (20.0)	2 (40.0)	1 (20.0)	1 (20.0)	0 (0.0)	0 (0.0)	2 (40.0)	1 (20.0)	1 (20.0)	3 (60.0)	1 (20.0)	0 (0.0)	1 (20.0)	0 (0.0)
***Shigella Spps*. (n = 2)**	0 (0.0)	0 (0.0)	0 (0.0)	0 (0.0)	NA	1 (50.0)	0 (0.0)	0 (0.0)	0 (0.0)	1 (50.0)	0 (0.0)	NA	1 (50.0)	0 (0.0)	NA	0 (0.0)	0 (0.0)
**Salmonella spps.(n = 2)**	0 (0.0)	NA	NA	1 (50.0)	NA	1 (50.0)	0 (0.0)	1 (50.0)	0 (0.0)	1 (50.0)	1 (50.0)	NA	2 (100)	NA	NA	0 (0.0)	NA
***M*. *morganii* (n = 4)**	3 (75.0)	2 (50.0)	3 (75.0)	3 (75.0)	NA	3 (75.0)	1 (25.0)	1 (25.0)	1 (25.0)	3 (75.0)	2 (50.0)	2 (50.0)	4 (100)	4 (100)	0 (0.0)	1 (25.0)	1 (25.0)
***Providencia* spps. (n = 6)**	1 (16.7)	0 (0.0)	0 (0.0)	2 (33.3)	4 (66.7)	1 (16.7)	0 (0.0)	1 (16.7)	2 (33.3)	2 (33.3)	1 (16.7)	1 (16.7)	6 (100)	5 (83.3	1 (16.7)	2 (33.3)	1 (16.7)
**Serratia spp. (n = 3)**	1 (33.3)	1 (33.3)	0 (0.0)	2 (66.7)	3 (100)	2 (66.7)	0 (0.0)	0 (0.0)	1 (33.3)	1 (33.3)	2 (66.7)	1 (33.3)	3 (100)	3 (100)	1 (33.3)	2 (66.7)	1 (33.3)
**Total Resistance (n = 338)**	**152 (44.9)**	**149 (44.1)**	**154 (45.6)**	**162 (47.9)**	**163 (48.2)**	**49 (14.5)**	**9 (2.7)**	**11 (3.3)**	**14 (4.1)**	**188 (55.6)**	**136 (40.2)**	**72 (21.3)**	**255 (75.4)**	**216 (64.0)**	**7 (2.1)**	**163 (48.2)**	**34 (10.1)**

### Multi-drug resistance pattern of gram negative bacilli

Using the commonly used definition of multidrug resistance (MDR) as an organism being resistant to three or more classes of antibiotics, 74.0% (n = 250) of the 338 isolates were identified as MDR. A high average MDR level was recorded among *Enterobacter* spp.(90.9%,), *Citrobacter* spp.(90.0%), *Acinetobacter* spp.(88.9%), *K*. *pneumoniae* (82.9%)and *E*. *coli* (69.6%). While *Pseudomonas* spp. showed a 100% MDR level, no MDR was found in the *K*. *oxytoca* and *K*. *ozaenae* isolates. The most effective antibiotics for MDR Gram negative bacilli were amikacin (97.1%), meropenem (96.3%), and imipenem (95.5%) **(Table 3)**.

**Table 3:** Multidrug resistance pattern of gram negative bacilli.

Level of antibiotics resistance (n (%))									
Isolates(number)	R0	R1	R2	R3	R4	R5	R6	≥R7	Total MDR isolates
*E*.*coli* (224)	28(12.5)	18(8.0)	22(9.8)	33(14.7)	12(5.4)	29(12.9)	33(14.7)	49(21.9)	**156(69.6)**
*K*.*pneumoniae* (41)	2(4.9)	3(7.3)	2(4.9)	6(14.6)	2(4.9)	2(4.9)	9(22.0)	15(36.6)	**34(82.9)**
*K*.*oxytoca* (4)	2(50.0)	2(50)	0(0.0)	0(0.0)	0(0.0)	0(0.0)	0(0.0)	0(0.0)	**0(0.0)**
*K*.*ozanea* (1)	1(100)	0(0.0)	0(0.0)	0(0.0)	0(0.0)	0(0.0)	0(0.0)	0(0.0)	**0(0.0)**
*Pseudomonas* spp.(17)	0(0.0)	0(0.0)	0(0.0)	2(11.8)	1(5.9)	0(0.0)	4(23.5)	10(58.8)	**17(100)**
*Enterobacter* spp.(11)	0(0.0)	0(0.0)	1(9.1)	0(0.0)	2(18.2)	0(0.0)	1(9.1)	7(63.7)	**10(90.9)**
*Citrobacter* spp.(10)	0(0.0)	0(0.0)	1(10.0)	2(20.0)	2(20.0)	1(10.0)	4(40.0)	0(0.0)	**9(90.0)**
*Acinetobacter* spp.(9)	0(0.0)	0(0.0)	1(11.1)	3(33.3)	1(11.1)	1(11.1)	1(11.1)	2(22.2)	**8(88.9)**
*P*.*mirabilis* (5)	2(40.0)	0(0.0)	1(20.0)	0(0.0)	1(20.0)	0(0.0)	1(20.0)	0(0.0)	**2(40.0)**
*Shigella* spp.(2)	1(50.0)	0(0.0)	0(0.0)	0(0.0)	0(0.0)	1(50.0)	0(0.0)	0(0.0)	**1(50.0)**
*Salmonella* spp.(2)	1(50.0)	1(50.0)	0(0.0)	0(0.0)	0(0.0)	0(0.0)	0(0.0)	0(0.0)	**0(0.0)**
*M*. *morganii* (4)	2(50.0)	1(25.0)	0(0.0)	1(25.0)	0(0.0)	0(0.0)	0(0.0)	0(0.0)	**1(25.0)**
*Prividencia* spp.(6)	1(16.7)	3(50.0)	0(0.0)	1(16.7)	0(0.0)	1(16.7)	0(0.0)	0(0.0)	**2(33.3)**
*Serratia* spp.(3)	1(33.3)	2(66.7)	0(0.0)	0(0.0)	0(0.0)	0(0.0)	0(0.0)	0(0.0)	**0(0.0)**
**Total (n = 338)**	**35(11.0)**	**24(7.1)**	**29(8.6)**	**49(14.5)**	**23(6.8)**	**36(10.6)**	**54(16.0)**	**88(26.0)**	**250(74.0)**

Note: R0: no resistance to antibiotics, R1-7: resistance to 1, 2, 3, 4, 5, 6, and 7 antibiotics respectively; MDR: A bacterium that is simultaneously resistant to three or more antibiotics from different antibiotic classes.

### Magnitude of ESBLs producing gram negative bacilli

From the total 338 isolated GNB, 135 were found to be suspicious for ESBLs production based on their MIC value of ≥2μg/ml for ceftazidime and/or cefotaxime as obtained from Phoenix system. Of these 135 GNB, 38.8% (131/338) of them were finally confirmed as ESBLs producers using the confirmatory combination disk method.

**Fig 1:**
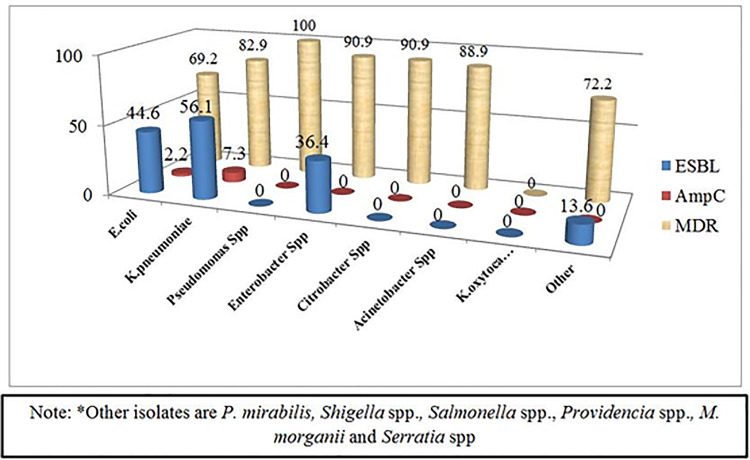
Distribution of ESBLs and AmpC beta-lactamases producing GNB and MDR level among GNB.

A higher percentage of ESBL producing GNB was identified in *K*. *pneumoniae* 56.1% (n = 23/41) followed by *E*. *coli (*44.6%, 100/224), *E*. *cloacae* (36.4%, 4/11) and *Citrobacter spp*. (10.0%, 1/10) **(Fig 1)**. The majority of these ESBLs producing GNB were detected in urine specimens and the proportion of ESBLs was significantly high among isolates from adult patients above 61 years of age (p < 0.05).

### Magnitude of AmpC producing gram negative bacilli

All ESBLs producers and cefoxitin resistant GNB were screened and confirmed for AmpC production. Of all tested Gram negative bacilli, 2.4% (8/338) were found to be AmpC beta-lactamase producers which are *E*. *coli* in 1.2% (4/338), *K*. *pneumoniae* in 0.9% (3/338) and *Citrobacter spp*. in 0.3% (1/338).

The highest frequency of AmpC beta-lactamase production was observed from *K*. *pneumoniae* 7.3% (3/41) followed by *E*. *coli* 1.8% (4/224).

Among the 80 GNB isolates that showed resistance to cefoxitin (MIC >16 μg/ml), 10% (8/80) of them were confirmed as AmpC producers. All AmpC beta-lactamase producing GNB were detected in urine specimens 3.3% (8/244) and in patients whose age group was above 61 years (40.1%).

The MDR level of each Gram negative bacillus in relation to their ESBLs and AmpC production is summarized below **(Fig 1).**

### Distribution of ESBLs and AmpC beta-lactamase producing gram negative bacilli with their MDR level among different specimens

From the specimens analyzed, ESBLs and/or AmpC producing GNB were found predominantly in urine 44.7% (109/244). The highest number of MDR isolates were found in wound specimens (81.0%, n = 51/249) (**Table 4**). Of ESBLs and/or AmpC producing GNB, 97.8% (n = 137/140) were MDR and only 1.4% (n = 2/140) was non-beta-lactamases producing MDR Gram negative bacilli due to other resistance mechanisms. Being beta-lactamases producer has statistically significant association with the occurrence of MDR (p = 0.001).

**Table 4:** Distribution of ESBLs, AmpC beta-lactamase producing GNB and MDR isolates from different clinical specimens.

Specimens(number)	Types of beta-lactamase n (%)		
	ESBL	AmpC	MDR
Urine(244)	107(44.0)	7(3.3)	181(74.2)
Pus(63)	21(33.3)	0(0.0)	51(81.0)
Body fluid(10)	1(0.1)	1(0.1)	5(50.0)
Other specimens* (21)	2(9.5)	0(0.0)	12(57.1)
**Total(n = 338)**	**131(38.8)**	**8(2.4)**	**249(73.7)**

Note

* Other specimens: Sputum (6), stool (4), ear and eye discharge (11).

### Association of independent variables with magnitude of ESBLs and AmpC producing gram negative bacilli

Analysis of data using logistic regression model showed that the magnitude of ESBLs or AmpC producing GNB had statistically significant association with age group and specimen type. However, there was no statistical significance between sex and health facilities for acquisition of beta-lactamase producing GNB (P>0.05). GNB isolate that are isolated from age group >61year are (95%, AOR = 1.151 (0.055–0.413), p = 0.001) times more likely to be ESBLs or AmpC producer than other age group. The chances of getting beta-lactamase positive among GNB which are isolated from urine specimens are (AOR = 8.015 (1.378, 46.630), P = 0.021) times higher than beta-lactamase producing GNB isolated from pus, body fluid and other specimen.

### Antibiotics sensitivity pattern of ESBLs and AmpC producing gram negative bacilli

**Fig 2:**
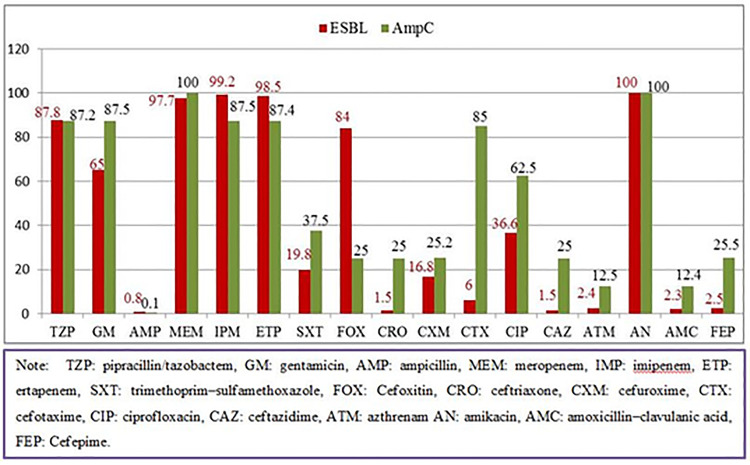
Antibiotics susceptibility pattern of ESBLs and AmpC producing GNB to different classes of antibiotics.

Overall non-ESBLs and/or AmpC producing GNB were more sensitive to tested antibiotics than ESBLs and/or AmpC producers. The most active drugs for ESBLs producing isolates were amikacin (100%), imipenem (99.2%), ertapenem (98.5%), meropenem (97.7%) and pipracillin/tazobactam (87.8%). Similarly, AmpC producing GNB showed high sensitivity to amikacin (100%), meropenem (100%), imipenem (87.5%), ertapenem (87.4%) and pipracillin/tazobactam (87.2%) (Fig 2).

## Discussion

### Antibiotics resistance pattern of gram negative bacilli

In the present study, the level of antimicrobial resistance of gram negative bacilli ranged from 0–75.4%. A higher resistance level was seen to ampicillin (75.4%), amoxicillin with clavulanic acid (64.0%), sulfamethoxazole-trimethoprim (55.6%), aztreonam and cefuroxime (48.8%), cefotaxime (47.0%), cefepime (45.6%), ceftriaxone (44.9%) and ceftazidime (44.1%). Comparable results were reported from studies performed in Gondar [16] and Debre Markos [21] in Ethiopia. Compared to findings done in Tanzania [22], Iran [23] and Sierra Leon [24] that reported a resistance level of >90% for ampicillin, amoxicillin with clavulanic acid, sulfamethoxazole-trimethoprim and ceftazidime, the resistance level recorded for these antibiotics in our study was a lowered finding that was <76%. Possible reasons for this difference can be the methods employed for antimicrobial testing, indiscriminate usage of antibiotics, patients condition and the nature of bacteria in these countries that were beta-lactamases producing bacteria in most cases.

### Multi drug resistance pattern of gram negative bacilli

In the present study, the overall magnitude of MDR among all GNB isolates was 73.7%. There were similar results found in studies conducted in Gondar (68.0%) [25], Dessie (74.6%) [26] and Debre Markos: (72.2%) [21] in Ethiopia and also in Nepal (64.0%) [27]. Our study found a lowered MDR level than studies done in Gondar (87.4%) [16] and Bahir Dar (93.1%) [28] in Ethiopia and studies performed in Sierra Leone (85.7%) [24] and Nepal (96.8%) [29]. However, the magnitude of MDR we found among the GNB was higher than in previous studies done in Ethiopia (Jimma: 59.3%) [30] and Nepal (54.2%) [27]. The MDR level recorded in this study was considered as alarming because only a few treatment options remain for infections. Therefore, implementing strong infection control strategies is required to reduce the MDR burden.

The present study showed that *Pseudomonas spp*.(100%), *Enterobacter* spp. (90.0%) and *Citrobacter* spp (90.0%) were the commonest MDR isolates which is comparable to other studies done in Jimma in Ethiopia, 100% *Citrobacter* spp. [30] and in Nepal, 71.4% *Enterobacter* spp. [31]. Different studies showed different pathogens as predominant MDR isolates, *K*. *pneumoniae* (95.6%) and *E*. *coli* (92.9%) in Gondar in Ethiopia [16], *K*. *pneumoniae* (73.3%) and *E*. *coli* (61.5%) in Sierra Leone [23] and *K*. *pneumoniae* 100% and *E*. *coli* 95.5% in Nepal [29]. These pathogens are found in both hospital and community acquired infections. In addition, these bacteria are resistant to multiple groups of antimicrobial agents which makes treatment difficult [27].

### Magnitude of ESBLs producing gram negative bacilli

The overall magnitude of ESBLs producing GNB in the present study was 38.8%, which was in line with studies reported from Ethiopia in Harar (33.3%) [32], Nepal (34.5%) [27], Spain (42.8%) [33] and India (44.0%) [34]. ESBLs producing organisms are the major cause of treatment failure, impaired clinical and microbiological responses, longer hospital stay and increased healthcare costs [35].

However, the magnitude of ESBLs production among GNB in our study was lower than in other studies done in Addis Ababa in Ethiopia (57.7%) [36], Bahir Dar in Ethiopia (57.6%) [28] and north West Nigeria (58.0%) [37], southwest Uganda (89%) [38] and southeast Iran (53.8%) [23]. This wide variation might be due to differences in study population, type of specimen, sample size and the extent of antibiotic use.

To the contrary, our finding was higher than compared with other studies findings in Ethiopia from Adama (25.0%) [39], Nigeria (15.8%) [40], Nepal (26.8%) [29] and Italy (6.3%) [41]. This indicated that ESBLs-producing organisms are increasing from time to time.

In the current study, the predominant ESBLs producing isolates were *K*. *pneumoniae* (56.1%) and *E*. *coli* (43.8%). This finding was supported by previous studies done in Ethiopia in Addis Ababa: *K*. *pneumoniae* in 78.6% and *E*. *coli* in 52.2% [36] and in Bahir Dar: *K*. *pneumoniae* in 69.8% and *E*. *coli* in 58.2% [28]. Researches in other African countries also isolated these bacteria as predominant ESBLs. The previously mentioned study in north west Nigeria identified: *K*. *pneumoniae* in 62.9% and *E*. *coli* in 54.2% [37], the study in southwest Uganda: *K*. *pneumoniae* in 52% and *E*. *coli in* 44% [38], a study in Nairobi, Kenya: *K*. *pneumoniae* in 78.8% and *E*. *coli* in 60.7% [42], a study done in Uganda: *K*. *pneumoniae* in 72.7% and *E*. *coli* in 58.1% [43]. On the other side, our finding was in contrast with studies conducted in Sri Lanka: *E*. *coli* in 86.8% and *K*. *pneumoniae* in 13.1% [44] and India: *E*. *coli* in 50.14% and *K*. *pneumoniae* in 48.3% [45]. In these studies, *E*. *coli* was the predominant ESBLs producer instead of *K*. *pneumoniae*.

### Magnitude of AmpC producing gram negative bacilli

AmpC beta-lactamase producing GNB have been responsible for several nosocomial outbreaks and high rate of treatment failure [46].

In the present study, the magnitude of AmpC producing GNB was 2.4% (8/338). Studies done in Iran (1.5%) [47], Greek (2.6%) [48] and India (8%) [49] showed equivalent numbers. However, studies from Nigeria (15.2%) [46], Spain (14.2%) [33] and India (37%) [34] showed a higher number of AmpC-producing GNB. Reasons for these different findings might be related to detection methods employed, study participants, geographic area and AmpC genes prevalence difference.

In present study, *K*. *pneumoniae* (7.3%, 3/41) and *E*. *coli* (2.2%, 5/224) were the principal AmpC producing GNB. This was also found in a study conducted in Turkey [50] and Spain [33]. This might be related to the fact that plasmid mediated AmpC beta-lactamases are seen in *Enterobacteriaceae* and these genes are easily horizontally transferred [13].

The current study also demonstrated co-existence of ESBLs and AmpC enzymes in five isolates 3.6% (5/139). This finding was comparable with studied conducted in Nigeria (6.04%) [40], South India (4.4%) [2] and India (9.9%) [9]. Simultaneous production of ESBLs and AmpC enzymes in a bacterium causes false-negative confirmatory tests for ESBLs production, because existence of plasmid mediated AmpC beta-lactamase enzymes can mask the presence of ESBLs [51]. Therefore, simultaneous detection of these enzymes is important to prevent the chance of missing an ESBLs.

### Antibiotic susceptibility pattern of ESBLs and AmpC producing gram negative bacilli

In the present study, the highest sensitivity level of ESBLs producing isolates was found to amikacin (100%), imipenem (99.2%), meropenem (97.7%), ertapenem (98.5%), piperacillin/tazobactam (87.0%) and cefoxitin (84.0%). This finding was in congruent with findings of other studies conducted in Ghana: 100% sensitive to meropenem [52], Nepal: imipenem (100%), piperacillin/tazobactam (93.3%), and amikacin (91.8%) [27], Sri Lanka: meropenem (95%), imipenem (73.7%) and amikacin (60.6%) [44] and India: imipenem (100%), piperacillin/tazobactam (89.3%), meropenem (87.5%), and amikacin (83.9%) [45].

The present study indicated that ESBLs producers had significant levels of resistance to third generation cephalosporines, penicillines and sulfonamides. Similar findings were observed in studies from Ethiopia (Adama), Uganda and Ghana [39, 43, 52]. Highest levels of resistance to ampicillin (99.2%), ceftazidime (98.5%), ceftriaxone (98.5%), amoxicillin with clavulanic acid (98.0%) and sulfamethoxazole-trimethoprim (81.0%) observed in this study were also reported in other studies done in Nepal: amoxicillin with clavulanic acid (100%), India: ceftazidime (97%), ceftriaxone (76%) [53] and another study in India done by Shashwati *et al*, amoxicillin with clavulanic acid (89.3%), sulfamethoxazole-trimethoprim (94.6%) [45].

Most of the AmpC producing GNB were resistant to the commonly used antibiotics which are known from several studies [40, 51, 54]. In our study, among cefoxitin resistance isolates, 10% (8/80) were AmpC producers which was in agreement with findings from studies done in Iran (5.1%) [47] and Turkey (8.7%) [50] but lower than findings from a study done in India (37.0%) [53]. The possible reasons for cefoxitin resistance in the absence of AmpC production might be due to other resistance mechanism like loose of permeability of porins [55].

The present study showed that AmpC producers were 100% sensitive to imipenem and meropenem (100%) but highly resistant to ampicillin (100%), amoxicillin with clavulanic acid (97.0%), ceftriaxone (89.0%), sulfamethoxazole-trimethoprim (75.0%), ceftazidime (68.0%), and cefepime (50.0%). This finding was in line with a study conducted in Turkey which showed that AmpC producers were 100% sensitive to imipenem and meropenem, 92.0% to amikacin and that they were in 82.0% resistance to amoxicillin with clavulanic acid in 68.0% to ceftazidime and in 49.0% to cefepime [50], Likewise, AmpC producers were resistant to amoxicillin with clavulanic acid (77.9%) and ceftazidime (75.0%) in a Nigeria study [46] and to amoxicillin with clavulanic acid was (95.9%), sulfamethoxazole-trimethoprim (82.9%), and ceftazidime (87.1%) in an Indian study [53]. AmpC producers seem susceptible to cephalosporins in-vitro but when cephalosporins are used in vivo, they result in treatment failure [56, 57]. Therefore, cephalosporins are not useful in treating infections caused by AmpC producing bacteria.

### Distribution of ESBLs and AmpC producing gram negative bacilli in different specimens

The present study shows that ESBLs and/or AmpC producing GNB were predominantly found in urine (44.7%, 109/244) and in pus (34.9%, 22/63). This might be due to the larger number of urine and pus samples included in this study. In this study most ESBLs producers were found in urine. Similar findings were reported in several countries: northwest of Nigeria (63.5%) [37], Uganda 64.9% [43], Ghana 66.7% [52], Sierra Leone 64.3% [24] and India (52.3% and 35%) [45, 53]. Another study done in Adama, Ethiopia found pus as the major source of ESBLs producing GNB (53.0%) [39]. This difference is most likely related to the difference in the proportion of clinical specimens included, nature of study participants difference and risk factors.

All AmpC producing pathogens in our study were isolated from urine. Studies conducted in Turkey and Nigeria [50, 57] had equivalent findings. Isolates from sputum (50.0%) were the predominant producers of AmpC beta-lactamase in a study done in Nigeria by Ogefere *et al* [46]. This indicated that the prevalence of AmpC beta-lactamase may differs significantly among bacteria obtained from different clinical specimens [34].

## Conclusion and recommendation

In this study, a higher magnitude of ESBLs and AmpC beta-lactamases producing GNB was found. Majority of these beta-lactamase producing isolates were isolated from urine specimen. *K*. *pneumoniae* and *E*. *coli* were the most frequent identified ESBLs and AmpC beta-lactamase producing gram negative bacilli. These ESBLs and AmpC beta-lactamases producing isolates showed a high MDR level and were resistant for most available antibiotics. The most effective antibiotics for treatment of the identified gram negative ESBLs and AmpC beta-lactamases producers were amikacin, meropenem and imipenem. The occurrence of ESBLs and AmpC beta-lactamases requires strengthening of the antimicrobial resistance surveillance system and an effective antibiotic policy involving antibiotic restriction, combination therapy and infection control programs combined with good medical practices. Additionally, large scale studies that can assess the magnitude of ESBLs and AmpC beta-lactamases producers from a wider geographical perspective with more representative samples need to be done in the country.

### Limitation of the study

- The magnitude of ESBLs and AmpC producing GNB from blood culture was not addressed because of service interruption due to down time of the Bactech 9050 machine during the study period.

## Supporting information

S1 Data Raw data of the study. (PDF)

S2 Data Survey of extended-spectrum beta-lactamase and AmpC beta-lactamases producing gram negative bacilli isolated from clinical specimens at International Clinical Laboratories, Addis Ababa, Ethiopia. (DOCX)

## List of abbreviations

CLSI: Clinical and Laboratory Standards Institute
ICL: International Clinical Laboratories
ESBLs: Extended-spectrum beta-lactamases
AmpC: AmpC beta-lactamase
MDR: Multidrug Resistance
GNB: Gram negative bacilli
AST: Antibiotic Susceptibility Testing
EUCAST: European Committee on Antimicrobial Susceptibility Testing
MIC: Minimum Inhibitory Concentration
ATCC: American Type Culture Collection
