# Supplementary material for: Extended-spectrum Beta-lactamase and AmpC beta-lactamases producing gram negative bacilli isolated from clinical specimens at International Clinical Laboratories, Addis Ababa, Ethiopia
Source: PLoS One. 2020 Nov 12;15(11):e0241984. doi: 10.1371/journal.pone.0241984 (PMC7660541; doi:10.1371/journal.pone.0241984)
Supplement: S1 Data — (PDF) [file pone.0241984.s001.pdf]

Row Data.sav

	ID	Age	AgeGroup	Sex	Specimen	GNB
1	327	32	32-46	Female	Urine	E.coli
2	902	80	>=61	Female	Urine	K.pneumoniae
3	108	47	46-61	Male	Urine	E.coli
4	270	35	32-46	Female	Other	K.pneumoniae
5	272	35	32-46	Female	Other	Pseudomonas Spp
6	161	84	>=61	Female	Urine	E.coli
7	102	30	16-32	Female	Urine	E.coli
8	272	28	16-32	Female	Other	Pseudomonas Spp
9	119	13	<=15	Female	Other	P.mirabilis
10	115	68	>=61	Female	Urine	E.coli
11	588	31	16-32	Female	Urine	E.coli
12	301	50	46-61	Female	Urine	E.coli
13	141	1	<=15	Female	Urine	E.coli
14	197	22	16-32	Female	Urine	K.pneumoniae
15	165	11	<=15	Female	Urine	E.coli
16	682	42	32-46	Female	Urine	E.coli
17	286	70	>=61	Male	Urine	E.coli
18	321	49	46-61	Male	Other	Pseudomonas Spp
19	213	36	46-61	Male	Body fluid	E.coli
20	216	36	46-61	Male	Body fluid	Other
21	574	22	16-32	Female	Urine	E.coli
22	209	36	32-46	Female	Urine	E.coli
23	996	75	>=61	Male	Urine	E.coli
24	294	28	16-32	Female	Urine	K.pneumoniae
25	546	55	46-61	Male	Wound	E.coli
26	582	52	46-61	Female	Urine	K.pneumoniae
27	596	52	46-61	Female	Urine	Other
28	288	53	46-61	Female	Urine	Pseudomonas Spp
29	858	56	46-61	Female	Urine	K.pneumoniae
30	214	71	>=61	Female	Urine	E.coli
31	805	23	16-32	Female	Urine	Acinetobacter Spp
32	963	86	>=61	Male	Urine	E.coli
33	742	30	16-32	Male	Wound	Enterobacter Spp
34	740	69	>=61	Female	Urine	Enterobacter Spp
35	801	40	32-46	Male	Urine	E.coli

	BetaLactamase	ESBLPhoenix	ESBL Manual	CTX	CRO	CAZ	FEP	ATM	CXM	XM	AMP	AMC
1	ESBL	POS	POS	R	R	R	R	R	R	R	R	R
2	NEG	NEG	NEG	S	S	S	S	S	S	S	R	R
3	ESBL	POS	POS	R	R	R	R	R	R	R	R	R
4	NEG	NEG	NEG	S	S	S	S	S	S	S	R	R
5	NEG	NEG	NEG	R	R	S	S	S	R	S	R	R
6	NEG	NEG	NEG	R	S	S	S	S	S	S	R	R
7	NEG	NEG	NEG	S	S	S	S	S	S	S	S	S
8	NEG	NEG	NEG	R	R	S	S	S	R	S	R	R
9	NEG	NEG	NEG	S	S	S	S	S	S	S	R	S
10	ESBL	POS	POS	R	R	R	S	R	R	R	R	R
11	NEG	NEG	NEG	S	S	S	S	S	S	S	S	S
12	ESBL	POS	POS	R	R	R	R	R	R	R	R	R
13	ESBL	POS	POS	R	R	R	R	R	R	R	R	R
14	NEG	NEG	NEG	S	S	S	S	S	S	S	R	S
15	ESBL	POS	POS	R	R	R	R	R	R	R	R	R
16	ESBL	POS	POS	R	R	R	R	R	R	R	R	R
17	ESBL	POS	POS	R	R	R	R	R	R	R	R	R
18	NEG	NEG	NEG	R	R	S	S	S	R	S	R	R
19	ESBL	POS	POS	R	R	R	R	S	R	S	R	R
20	NEG	NEG	NEG	R	R	S	S	S	R	S	R	R
21	NEG	NEG	NEG	S	S	S	S	S	S	S	S	S
22	NEG	NEG	NEG	S	S	S	S	S	S	S	S	S
23	NEG	NEG	NEG	S	S	S	S	S	S	S	S	S
24	NEG	NEG	NEG	S	S	S	S	S	R	S	R	S
25	ESBL	POS	POS	R	R	R	R	R	R	R	R	R
26	NEG	NEG	NEG	S	S	S	S	S	S	S	R	S
27	ESBL	POS	POS	R	R	R	R	S	S	S	R	R
28	NEG	NEG	NEG	S	S	S	S	S	S	S	R	R
29	NEG	NEG	NEG	S	S	S	S	S	S	S	R	S
30	NEG	NEG	NEG	R	S	S	S	S	S	S	R	R
31	NEG	NEG	NEG	R	R	S	R	S	R	S	R	R
32	ESBL	POS	POS	R	R	R	R	R	R	R	R	R
33	ESBL	POS	NEG	R	R	S	S	R	R	R	R	R
34	ESBL	POS	POS	R	R	S	R	R	R	R	R	R
35	ESBL	POS	POS	R	R	R	R	R	R	R	R	R

	FOX	MEM	IMP	ETP	SXT	CIP	NOR	GM	AN	TZP	MDR2	MDR3	AmpC
1	S	S	S	S	S	R	S	R	S	S	R5	YES	NEG
2	S	S	S	S	S	R	S	S	S	S	RO	NO	NEG
3	S	S	S	S	R	R	R	R	S	S	R5	YES	NEG
4	S	S	R	S	S	R	S	S	S	S	R3	YES	NEG
5	S	S	S	R	R	R	R	R	S	S	R7	YES	NEG
6	S	S	S	S	R	S	S	S	S	S	R3	YES	NEG
7	S	S	S	S	S	S	S	S	S	S	RO	NO	NEG
8	S	S	S	R	R	S	S	S	S	S	R6	YES	NEG
9	S	S	S	S	R	S	S	S	S	S	R2	NO	NEG
10	S	S	S	S	S	R	R	S	S	S	R6	YES	NEG
11	S	S	S	S	S	S	S	S	S	S	RO	NO	NEG
12	S	S	S	S	R	R	S	S	S	S	R6	YES	NEG
13	S	S	S	S	S	R	S	S	S	S	R5	YES	NEG
14	S	S	S	S	S	S	S	S	S	S	R1	NO	NEG
15	S	S	S	S	S	R	S	S	S	S	R5	YES	NEG
16	S	S	S	S	R	R	S	R	S	S	R6	YES	NEG
17	S	S	S	S	R	R	R	R	S	S	R7	YES	NEG
18	S	R	S	R	R	R	R	S	R	S	R8	YES	NEG
19	S	S	S	S	R	R	S	S	S	S	R5	YES	NEG
20	S	S	S	S	R	R	S	R	R	S	R7	YES	NEG
21	S	S	S	S	S	S	S	S	S	S	RO	NO	NEG
22	S	S	S	S	S	S	S	S	S	S	RO	NO	NEG
23	S	S	S	S	S	S	S	S	S	S	RO	NO	NEG
24	S	S	S	S	R	S	S	S	S	S	R3	YES	NEG
25	S	S	S	S	R	R	R	S	S	S	R7	YES	NEG
26	S	S	S	S	S	S	S	R	S	S	RO	NO	NEG
27	S	S	S	S	R	S	S	R	S	S	R4	YES	NEG
28	S	S	S	R	R	S	S	S	S	S	R4	YES	NEG
29	S	S	S	S	S	S	S	S	S	S	R1	NO	NEG
30	S	S	S	S	S	S	S	S	S	S	R2	NO	NEG
31	R	S	S	R	S	S	S	S	S	S	R6	YES	NEG
32	S	S	S	S	S	R	R	S	S	S	R6	YES	NEG
33	R	S	S	S	S	S	S	S	S	S	R6	YES	NEG
34	R	S	S	S	R	S	S	R	S	S	R7	YES	NEG
35	S	S	S	S	R	R	S	R	S	S	R6	YES	NEG

	BLCONFIRMATORY	GNBPOS	Healthcenter
1	POS	POS	Governmen
2	NEG	POS	Governmen
3	POS	POS	Private
4	NEG	POS	Private
5	NEG	POS	Private
6	NEG	POS	Governmen
7	NEG	POS	Governmen
8	NEG	POS	Private
9	NEG	POS	Governmen
10	POS	POS	Private
11	NEG	POS	Private
12	POS	POS	Governmen
13	POS	POS	Governmen
14	NEG	POS	Governmen
15	POS	POS	Private
16	POS	POS	Private
17	POS	POS	Governmen
18	NEG	POS	Private
19	POS	POS	Governmen
20	NEG	POS	Private
21	NEG	POS	Governmen
22	NEG	POS	Private
23	NEG	POS	Governmen
24	NEG	POS	Private
25	POS	POS	Governmen
26	NEG	POS	Private
27	POS	POS	Governmen
28	NEG	POS	Private
29	NEG	POS	Governmen
30	NEG	POS	Private
31	NEG	POS	Private
32	POS	POS	Governmen
33	POS	POS	Private
34	POS	POS	Private
35	POS	POS	Private

	ID	Age	AgeGroup	Sex	Specimen	GNB
36	285	80	>=61	Male	Urine	E.coli
37	857	4	<=15	Male	Wound	Other
38	861	4	<=15	Female	Wound	K.oxytoca & k.oz
39	807	4	<=15	Female	Wound	P.mirabilis
40	808	45	32-46	Male	Wound	Enterobacter Spp
41	604	40	32-46	Female	Urine	E.coli
42	212	56	46-61	Female	Urine	E.coli
43	213	56	46-61	Female	Urine	E.coli
44	816	85	>=61	Female	Urine	K.pneumoniae
45	922	7	<=15	Female	Urine	E.coli
46	607	44	32-46	Female	Urine	E.coli
47	125	27	16-32	Male	Body fluid	E.coli
48	224	27	16-32	Male	Body fluid	E.coli
49	760	80	>=61	Male	Other	K.pneumoniae
50	796	36	32-46	Female	Urine	K.pneumoniae
51	164	40	32-46	Female	Urine	Citrobacter Spp
52	338	3	<=15	Female	Urine	E.coli
53	341	30	16-32	Female	Urine	E.coli
54	839	13	<=15	Female	Urine	K.pneumoniae
55	152	50	46-61	Female	Urine	E.coli
56	700	28	16-32	Female	Urine	E.coli
57	129	30	16-32	Female	Urine	E.coli
58	335	32	32-46	Male	Other	Shigella Spp
59	133	60	46-61	Female	Wound	Pseudomonas Spp
60	233	71	>=61	Female	Wound	K.pneumoniae
61	229	35	32-46	Male	Body fluid	E.coli
62	230	35	32-46	Male	Body fluid	E.coli
63	115	1	<=15	Female	Urine	E.coli
64	950	55	46-61	Male	Wound	E.coli
65	347	29	16-32	Female	Wound	E.coli
66	199	32	32-46	Male	Wound	E.coli
67	933	39	32-46	Female	Urine	E.coli
68	198	28	16-32	Male	Wound	K.pneumoniae
69	206	68	>=61	Male	Urine	E.coli
70	314	54	46-61	Male	Body fluid	E.coli

	BetaLactamase	ESBLPhoenix	ESBL Manual	CTX	CRO	CAZ	FEP	ATM	CXM	XM	AMP	AMC
36	NEG	NEG	NEG	R	R	S	R	R	R	R	R	R
37	NEG	NEG	NEG	S	S	S	S	S	R	S	R	R
38	NEG	NEG	NEG	S	S	S	S	S	S	S	S	S
39	NEG	NEG	NEG	S	S	S	S	S	S	S	S	S
40	ESBL	POS	POS	R	R	R	R	R	R	R	R	R
41	NEG	NEG	NEG	S	S	S	S	S	S	S	S	S
42	ESBL	POS	POS	R	R	R	R	R	R	R	R	R
43	NEG	NEG	NEG	S	S	S	S	S	S	S	S	R
44	ESBL	POS	POS	R	R	R	R	R	R	R	R	R
45	NEG	NEG	NEG	S	S	S	S	S	S	S	S	S
46	NEG	NEG	NEG	S	S	S	S	S	S	S	S	S
47	NEG	NEG	NEG	S	S	S	S	S	S	S	R	R
48	NEG	NEG	NEG	S	S	S	S	S	S	S	R	R
49	NEG	NEG	NEG	S	S	S	S	S	S	S	R	R
50	ESBL	POS	POS	R	R	R	R	R	R	R	R	R
51	NEG	NEG	NEG	S	S	S	S	S	S	S	R	S
52	NEG	NEG	NEG	S	S	S	S	S	S	S	R	S
53	NEG	NEG	NEG	S	S	S	S	S	S	S	S	S
54	NEG	NEG	NEG	S	S	S	S	S	S	S	R	R
55	NEG	NEG	NEG	S	S	S	S	S	S	S	R	S
56	NEG	NEG	NEG	S	S	S	S	S	S	S	R	S
57	ESBL	POS	POS	R	R	R	R	R	R	R	R	R
58	NEG	NEG	NEG	S	S	S	S	S	S	S	S	S
59	NEG	NEG	NEG	R	R	S	S	S	R	S	R	R
60	ESBL	POS	POS	R	R	R	R	R	R	R	R	R
61	NEG	NEG	NEG	S	S	S	S	S	S	S	R	R
62	NEG	NEG	NEG	R	R	R	R	R	R	R	R	R
63	NEG	NEG	NEG	S	S	S	S	S	S	S	S	S
64	ESBL	POS	POS	R	R	R	R	R	R	R	R	R
65	NEG	NEG	NEG	S	S	S	S	S	S	S	R	R
66	NEG	NEG	NEG	S	S	S	S	S	S	S	R	S
67	NEG	NEG	NEG	S	S	S	S	S	S	S	S	S
68	ESBL	POS	POS	R	R	R	R	R	R	R	R	R
69	ESBL	POS	POS	R	R	R	R	R	R	R	R	R
70	NEG	NEG	NEG	R	R	R	R	R	R	R	R	R

	FOX	MEM	IMP	ETP	SXT	CIP	NOR	GM	AN	TZP	MDR2	MDR3	AmpC
36	S	S	S	R	R	R	S	R	S	S	R8	YES	NEG
37	S	S	S	R	S	S	S	S	S	S	R4	YES	NEG
38	S	S	S	S	S	S	S	S	S	S	RO	NO	NEG
39	S	S	S	S	S	S	S	S	S	S	RO	NO	NEG
40	R	S	S	S	R	R	S	R	S	S	R7	YES	NEG
41	S	S	S	S	S	S	S	S	S	S	RO	NO	NEG
42	R	S	S	S	S	S	S	S	S	S	R5	YES	POS
43	S	S	S	S	S	S	S	S	S	S	R1	NO	NEG
44	S	S	S	S	R	R	S	R	S	S	R6	YES	NEG
45	S	S	S	S	S	S	S	S	S	S	RO	NO	NEG
46	S	S	S	S	S	S	S	S	S	S	RO	NO	NEG
47	S	S	S	S	R	S	S	S	S	S	R3	YES	NEG
48	S	S	S	S	R	S	S	S	S	S	R3	YES	NEG
49	S	S	S	S	S	S	S	S	S	S	R2	NO	NEG
50	S	S	S	S	S	S	S	R	S	S	R5	YES	POS
51	S	S	S	S	R	R	S	S	S	S	R2	NO	NEG
52	S	S	S	S	R	S	S	S	S	S	R2	NO	NEG
53	S	S	S	S	S	S	S	S	S	S	RO	NO	NEG
54	R	S	S	S	R	S	S	S	S	S	R3	YES	POS
55	S	S	S	S	R	R	S	S	S	S	R3	YES	NEG
56	S	S	S	S	R	R	S	S	S	S	R3	YES	NEG
57	S	S	S	S	S	S	S	S	S	S	R5	YES	NEG
58	S	S	S	S	S	S	S	S	S	S	RO	NO	NEG
59	S	S	S	R	R	S	S	S	S	S	R6	YES	NEG
60	S	S	S	S	R	R	R	R	S	S	R7	YES	NEG
61	S	S	S	S	R	S	S	S	S	S	R3	YES	NEG
62	S	S	S	S	R	S	S	S	S	S	R6	YES	NEG
63	S	S	S	S	S	S	S	S	S	S	RO	NO	NEG
64	S	S	S	S	R	R	R	S	S	S	R7	YES	NEG
65	S	S	S	S	R	S	S	S	S	S	R3	YES	NEG
66	S	S	S	S	S	S	S	S	S	S	R1	NO	NEG
67	S	S	S	S	R	S	S	S	S	S	R1	NO	NEG
68	S	S	S	S	R	R	R	R	S	S	R7	YES	NEG
69	S	S	S	S	R	R	S	S	S	S	R6	YES	NEG
70	S	S	S	S	S	S	S	S	S	S	R5	YES	POS

	BLCONFIRMATORY	GNBPOS	Healthcenter
36	POS	POS	Governmen
37	NEG	POS	Private
38	NEG	POS	Governmen
39	NEG	POS	Private
40	POS	POS	Governmen
41	NEG	POS	Private
42	POS	POS	Governmen
43	NEG	POS	Private
44	POS	POS	Governmen
45	NEG	POS	Private
46	NEG	POS	Governmen
47	POS	POS	Private
48	NEG	POS	Governmen
49	NEG	POS	Private
50	POS	POS	Private
51	NEG	POS	Private
52	NEG	POS	Private
53	NEG	POS	Private
54	POS	POS	Governmen
55	NEG	POS	Governmen
56	NEG	POS	Private
57	POS	POS	Governmen
58	NEG	POS	Private
59	NEG	POS	Private
60	POS	POS	Governmen
61	NEG	POS	Governmen
62	NEG	POS	Private
63	NEG	POS	Private
64	POS	POS	Governmen
65	NEG	POS	Governmen
66	NEG	POS	Governmen
67	NEG	POS	Private
68	POS	POS	Private
69	POS	POS	Private
70	POS	POS	Governmen

	ID	Age	AgeGroup	Sex	Specimen	GNB
71	320	80	>=61	Female	Urine	K.pneumoniae
72	113	80	>=61	Male	Urine	Enterobacter Spp
73	313	80	>=61	Male	Urine	Other
74	128	38	32-46	Male	Urine	E.coli
75	955	7	<=15	Female	Urine	Acinetobacter Spp
76	414	40	32-46	Male	Wound	Enterobacter Spp
77	413	30	16-32	Male	Wound	Acinetobacter Spp
78	717	27	16-32	Female	Other	Pseudomonas Spp
79	537	45	32-46	Female	Urine	E.coli
80	117	49	46-61	Female	Urine	E.coli
81	110	45	32-46	Male	Urine	E.coli
82	668	67	>=61	Female	Urine	E.coli
83	793	70	>=61	Male	Other	E.coli
84	496	75	>=61	Female	Urine	K.pneumoniae
85	422	76	>=61	Male	Urine	E.coli
86	716	72	>=61	Male	Wound	E.coli
87	564	25	16-32	Female	Urine	E.coli
88	147	67	>=61	Male	Urine	E.coli
89	467	43	32-46	Female	Urine	E.coli
90	484	75	>=61	Male	Urine	E.coli
91	800	53	46-61	Male	Wound	E.coli
92	855	28	16-32	Female	Urine	E.coli
93	210	48	46-61	Male	Urine	Other
94	201	4	<=15	Male	Urine	Citrobacter Spp
95	202	4	<=15	Male	Urine	Acinetobacter Spp
96	109	40	32-46	Male	Urine	K.pneumoniae
97	894	28	16-32	Female	Urine	E.coli
98	893	77	>=61	Female	Urine	E.coli
99	138	45	32-46	Female	Urine	E.coli
100	970	1	<=15	Female	Urine	E.coli
101	932	22	16-32	Male	Wound	Acinetobacter Spp
102	267	52	46-61	Male	Other	K.pneumoniae
103	522	54	46-61	Male	Wound	E.coli
104	521	82	>=61	Female	Urine	E.coli
105	584	48	46-61	Female	Wound	E.coli

	BetaLactamase	ESBLPhoenix	ESBL Manual	CTX	CRO	CAZ	FEP	ATM	CXM	XM	AMP	AMC
71	ESBL	POS	POS	R	R	R	R	R	R	R	R	R
72	ESBL	NEG	NEG	S	S	S	S	S	S	S	R	R
73	ESBL	POS	POS	S	S	S	S	R	R	R	R	R
74	NEG	NEG	NEG	R	R	R	R	R	R	R	R	R
75	NEG	NEG	NEG	S	S	S	S	S	S	S	R	R
76	NEG	NEG	NEG	R	R	R	R	R	R	R	R	R
77	NEG	NEG	NEG	R	R	R	R	R	R	R	R	R
78	NEG	NEG	NEG	R	R	R	R	R	R	R	S	R
79	NEG	NEG	NEG	S	S	S	S	S	S	S	S	S
80	NEG	NEG	NEG	S	S	S	S	S	S	S	R	R
81	NEG	NEG	NEG	S	S	S	S	S	S	S	R	R
82	ESBL	POS	POS	R	R	R	R	R	R	R	R	R
83	ESBL	POS	POS	R	R	R	R	R	R	R	R	R
84	ESBL	POS	POS	R	R	R	R	R	R	R	R	R
85	ESBL	POS	POS	R	R	R	R	R	R	R	R	R
86	ESBL	POS	POS	R	R	R	R	R	R	R	R	R
87	NEG	NEG	NEG	S	S	S	S	S	S	S	S	S
88	ESBL	POS	POS	R	R	R	R	R	R	R	R	R
89	ESBL	POS	POS	R	R	R	R	R	R	R	R	R
90	ESBL	POS	POS	R	R	R	R	R	R	R	R	R
91	NEG	NEG	NEG	S	S	S	S	S	S	S	S	R
92	NEG	NEG	NEG	S	S	S	S	S	S	S	R	S
93	NEG	NEG	NEG	S	S	R	S	R	R	R	R	R
94	NEG	NEG	NEG	R	R	R	S	R	R	R	R	R
95	NEG	NEG	NEG	S	S	S	S	S	S	S	R	R
96	ESBL	POS	POS	R	R	R	R	R	R	R	R	R
97	ESBL	POS	POS	R	R	R	R	R	R	R	R	R
98	ESBL	POS	POS	R	R	R	R	R	R	R	R	R
99	ESBL	POS	POS	R	R	R	R	R	R	R	R	R
100	NEG	NEG	NEG	S	S	S	S	S	S	S	S	R
101	NEG	NEG	NEG	R	R	R	R	R	R	R	R	R
102	ESBL	POS	POS	R	R	R	R	R	R	R	R	R
103	NEG	NEG	NEG	R	R	S	R	R	R	R	R	S
104	NEG	NEG	NEG	S	S	S	S	S	R	S	S	S
105	NEG	NEG	NEG	S	S	S	S	S	S	R	S	R

	FOX	MEM	IMP	ETP	SXT	CIP	NOR	GM	AN	TZP	MDR2	MDR3	AmpC
71	S	S	S	S	R	R	S	S	S	S	R6	YES	NEG
72	S	S	S	S	S	S	S	S	S	S	R2	NO	NEG
73	R	R	S	S	R	R	S	S	S	S	R7	YES	NEG
74	R	R	R	R	R	S	S	R	S	R	R9	YES	NEG
75	S	S	S	S	S	S	S	S	S	S	R2	NO	NEG
76	R	S	S	R	R	S	S	R	S	R	R10	YES	NEG
77	R	S	S	S	R	R	R	R	S	R	R10	YES	NEG
78	R	S	S	S	R	S	S	S	R	S	R7	YES	NEG
79	S	S	S	S	S	S	S	S	S	S	RO	NO	NEG
80	S	S	S	S	R	S	S	S	S	S	R3	YES	NEG
81	S	S	S	S	R	S	S	S	S	S	R3	YES	NEG
82	S	S	S	S	R	R	S	S	S	S	R6	YES	NEG
83	S	S	S	S	R	R	R	R	S	R	R9	YES	NEG
84	S	S	S	S	R	R	R	S	S	S	R7	YES	NEG
85	S	S	S	S	R	S	S	R	S	S	R6	YES	NEG
86	S	S	S	S	R	S	S	R	S	R	R8	YES	NEG
87	S	S	S	S	S	S	S	S	S	S	RO	NO	NEG
88	S	S	S	S	R	R	R	R	S	S	R7	YES	NEG
89	S	S	S	S	R	S	S	R	S	S	R6	YES	NEG
90	S	S	S	S	R	R	R	R	S	S	R7	YES	NEG
91	S	S	S	S	R	S	S	S	S	S	R2	NO	NEG
92	S	S	S	S	S	S	S	S	S	S	R1	NO	NEG
93	R	S	R	S	R	S	S	S	S	R	R9	YES	NEG
94	S	S	S	S	R	R	S	R	S	S	R6	YES	NEG
95	S	S	S	R	S	S	S	S	S	S	R3	YES	NEG
96	S	S	S	S	R	S	S	S	S	S	R6	YES	POS
97	S	S	S	S	R	R	R	S	S	S	R7	YES	NEG
98	S	S	S	S	R	R	S	R	S	S	R6	YES	NEG
99	S	S	S	S	S	S	S	S	S	S	R5	YES	NEG
100	S	S	S	S	R	S	S	S	R	S	R3	YES	NEG
101	R	S	S	R	S	R	R	S	R	R	R10	YES	NEG
102	S	R	R	R	R	R	R	R	S	R	R11	YES	NEG
103	S	S	S	S	R	R	R	S	S	S	R6	YES	NEG
104	S	S	S	S	S	S	S	S	S	S	R1	NO	NEG
105	S	S	S	S	R	R	R	S	S	S	R5	YES	NEG

	BLCONFIR MATORY	GNBPOS	Healthcenter
71	POS	POS	Governmen
72	POS	POS	Governmen
73	POS	POS	Private
74	NEG	POS	Governmen
75	NEG	POS	Governmen
76	NEG	POS	Private
77	NEG	POS	Governmen
78	NEG	POS	Private
79	NEG	POS	Governmen
80	NEG	POS	Private
81	NEG	POS	Private
82	POS	POS	Governmen
83	POS	POS	Private
84	POS	POS	Governmen
85	POS	POS	Private
86	POS	POS	Governmen
87	NEG	POS	Private
88	POS	POS	Governmen
89	POS	POS	Governmen
90	POS	POS	Private
91	NEG	POS	Governmen
92	NEG	POS	Private
93	NEG	POS	Governmen
94	NEG	POS	Private
95	NEG	POS	Governmen
96	POS	POS	Governmen
97	POS	POS	Private
98	POS	POS	Private
99	POS	POS	Private
100	NEG	POS	Governmen
101	NEG	POS	Governmen
102	POS	POS	Governmen
103	NEG	POS	Private
104	NEG	POS	Private
105	NEG	POS	Private

	ID	Age	AgeGroup	Sex	Specimen	GNB
106	729	20	16-32	Female	Body fluid	K.pneumoniae
107	432	89	>=61	Male	Urine	E.coli
108	433	80	>=61	Male	Urine	Citrobacter Spp
109	317	4	<=15	Female	Urine	E.coli
110	362	24	16-32	Female	Urine	E.coli
111	548	50	46-61	Female	Wound	Acinetobacter Spp
112	517	69	>=61	Male	Urine	Other
113	187	71	>=61	Male	Urine	E.coli
114	554	38	32-46	Female	Urine	E.coli
115	468	46	46-61	Female	Urine	E.coli
116	586	42	32-46	Male	Urine	E.coli
117	968	66	>=61	Male	Wound	E.coli
118	308	69	>=61	Male	Urine	K.pneumoniae
119	961	61	>=61	Male	Urine	E.coli
120	332	62	>=61	Male	Urine	E.coli
121	434	70	>=61	Male	Urine	E.coli
122	251	38	32-46	Female	Urine	E.coli
123	203	8	<=15	Female	Urine	E.coli
124	487	1	<=15	Female	Urine	Pseudomonas Spp
125	218	45	32-46	Male	Wound	Acinetobacter Spp
126	188	72	>=61	Male	Urine	Other
127	227	56	46-61	Female	Urine	E.coli
128	139	56	46-61	Female	Urine	E.coli
129	320	41	32-46	Female	Urine	E.coli
130	251	29	16-32	Female	Urine	E.coli
131	615	50	46-61	Female	Urine	K.pneumoniae
132	324	54	46-61	Female	Urine	E.coli
133	501	55	46-61	Female	Urine	Other
134	449	27	16-32	Female	Urine	E.coli
135	420	22	16-32	Female	Urine	E.coli
136	409	67	>=61	Male	Urine	E.coli
137	410	37	32-46	Female	Urine	E.coli
138	132	59	46-61	Male	Urine	E.coli
139	250	23	16-32	Male	Urine	E.coli
140	661	38	32-46	Female	Urine	E.coli

	BetaLactamase	ESBLPhoenix	ESBL Manual	CTX	CRO	CAZ	FEP	ATM	CXM	XM	AMP	AMC
106	ESBL	POS	POS	R	R	R	R	R	R	R	R	S
107	ESBL	POS	POS	R	R	R	R	R	R	R	R	R
108	NEG	NEG	NEG	S	S	S	S	R	S	R	R	S
109	NEG	NEG	NEG	S	S	S	S	R	S	R	R	S
110	ESBL	POS	POS	S	S	S	S	S	S	S	R	S
111	NEG	NEG	NEG	R	R	S	S	S	R	S	R	S
112	ESBL	POS	POS	R	R	R	R	R	S	R	R	R
113	ESBL	POS	POS	R	R	R	R	R	R	R	R	R
114	ESBL	POS	POS	S	S	R	S	S	S	S	R	R
115	ESBL	POS	POS	R	R	R	R	R	R	R	R	R
116	NEG	NEG	NEG	S	S	S	S	S	S	S	R	R
117	ESBL	POS	POS	R	R	R	R	R	R	R	R	R
118	ESBL	POS	POS	R	R	R	R	R	R	R	R	R
119	NEG	NEG	NEG	S	S	S	S	S	S	S	S	S
120	ESBL	POS	POS	R	R	R	R	R	R	R	R	R
121	ESBL	POS	POS	R	R	R	R	R	R	R	R	R
122	NEG	NEG	NEG	S	S	S	S	S	S	S	R	R
123	NEG	NEG	NEG	S	S	S	S	S	S	S	R	R
124	NEG	NEG	NEG	S	S	S	S	S	S	S	R	R
125	NEG	NEG	NEG	S	S	S	S	S	S	S	S	R
126	NEG	NEG	NEG	R	R	S	R	R	R	R	R	R
127	ESBL	POS	POS	R	R	R	R	R	R	R	R	R
128	NEG	NEG	NEG	S	S	S	S	S	S	S	R	S
129	NEG	NEG	NEG	S	S	S	S	S	S	S	R	R
130	NEG	NEG	NEG	S	S	S	S	S	S	S	S	S
131	ESBL	POS	POS	R	R	R	R	R	R	R	R	R
132	ESBL	POS	POS	R	R	R	R	R	S	R	R	R
133	NEG	NEG	NEG	S	S	S	S	S	S	S	R	S
134	NEG	NEG	NEG	S	S	S	S	S	S	S	S	S
135	NEG	NEG	NEG	S	S	S	S	S	S	S	S	S
136	ESBL	POS	POS	R	R	R	R	R	R	R	R	R
137	ESBL	POS	POS	R	R	R	R	R	S	R	R	R
138	NEG	NEG	NEG	S	S	S	S	S	S	S	R	S
139	ESBL	POS	POS	R	R	R	R	R	R	R	R	R
140	ESBL	POS	POS	R	R	R	R	R	R	R	R	R

	FOX	MEM	IMP	ETP	SXT	CIP	NOR	GM	AN	TZF	MDR2	MDR3	AmpC
106	S	S	S	S	R	R	R	R	S	R	R8	YES	NEG
107	S	S	S	S	R	S	S	S	S	S	R6	YES	NEG
108	S	S	S	S	R	R	S	R	S	S	R6	YES	NEG
109	S	S	S	S	R	R	S	R	S	S	R3	YES	NEG
110	S	S	S	S	R	S	S	S	S	S	R2	NO	NEG
111	S	S	S	S	R	S	S	S	S	S	R4	YES	NEG
112	S	S	S	S	R	R	R	S	S	S	R6	YES	NEG
113	S	S	S	S	S	R	S	R	S	S	R5	YES	NEG
114	S	S	S	S	R	S	S	R	S	S	R4	YES	NEG
115	S	S	S	S	R	S	S	S	S	S	R6	YES	NEG
116	S	S	S	S	R	R	R	S	S	S	R4	YES	NEG
117	S	S	S	S	S	R	S	S	S	S	R5	YES	NEG
118	S	S	S	S	R	R	R	S	S	S	R7	YES	NEG
119	S	S	S	S	S	S	S	S	S	S	RO	NO	NEG
120	S	S	S	S	R	R	R	S	S	S	R7	YES	NEG
121	S	S	S	S	R	R	R	R	S	R	R8	YES	NEG
122	S	S	S	S	R	R	S	S	S	S	R3	YES	NEG
123	S	S	S	S	S	S	S	S	S	S	R2	NO	NEG
124	S	S	S	R	S	S	S	S	S	S	R3	YES	NEG
125	R	S	S	R	S	S	S	S	S	S	R3	YES	NEG
126	S	S	R	R	S	R	R	R	R	R	R10	YES	NEG
127	S	S	S	S	R	S	S	S	S	S	R6	YES	NEG
128	S	S	S	S	R	S	S	S	S	S	R2	NO	NEG
129	S	S	S	S	S	S	S	S	S	S	R2	NO	NEG
130	S	S	S	S	S	S	S	S	S	S	RO	NO	NEG
131	S	S	S	S	R	S	S	S	S	S	R6	YES	NEG
132	S	S	S	S	R	S	S	R	S	S	R5	YES	NEG
133	S	S	S	S	R	S	S	S	S	S	R2	NO	NEG
134	S	S	S	S	S	R	S	R	S	S	R2	NO	NEG
135	S	S	S	S	S	S	S	S	S	S	RO	NO	NEG
136	S	S	S	S	R	S	S	S	S	S	R6	YES	NEG
137	S	S	S	S	R	S	S	S	S	S	R5	YES	NEG
138	S	S	S	S	S	S	S	S	S	S	R1	NO	NEG
139	S	S	S	S	S	R	R	S	S	S	R6	YES	NEG
140	S	S	S	S	R	R	S	S	S	S	R6	YES	NEG

	BLCONFIR MATORY	GNBPOS	Healthcenter
106	POS	POS	Private
107	POS	POS	Private
108	NEG	POS	Governmen
109	POS	POS	Private
110	POS	POS	Private
111	NEG	POS	Governmen
112	POS	POS	Private
113	POS	POS	Governmen
114	POS	POS	Private
115	POS	POS	Private
116	NEG	POS	Private
117	POS	POS	Private
118	POS	POS	Private
119	NEG	POS	Governmen
120	POS	POS	Governmen
121	POS	POS	Private
122	NEG	POS	Governmen
123	NEG	POS	Private
124	NEG	POS	Governmen
125	NEG	POS	Private
126	NEG	POS	Private
127	POS	POS	Governmen
128	NEG	POS	Private
129	NEG	POS	Governmen
130	NEG	POS	Governmen
131	POS	POS	Private
132	POS	POS	Private
133	NEG	POS	Private
134	NEG	POS	Governmen
135	NEG	POS	Governmen
136	POS	POS	Private
137	POS	POS	Private
138	NEG	POS	Private
139	POS	POS	Private
140	POS	POS	Governmen

	ID	Age	AgeGroup	Sex	Specimen	GNB
141	780	35	32-46	Male	Wound	K.pneumoniae
142	581	35	32-46	Male	Wound	E.coli
143	289	25	16-32	Male	Urine	K.oxytoca & k.oz
144	291	46	46-61	Female	Urine	E.coli
145	720	54	46-61	Male	Urine	E.coli
146	561	20	16-32	Female	Urine	Enterobacter Spp
147	111	60	46-61	Female	Wound	K.pneumoniae
148	101	60	46-61	Female	Wound	Pseudomonas Spp
149	551	34	32-46	Female	Urine	E.coli
150	163	70	>=61	Male	Urine	E.coli
151	990	6	<=15	Male	Wound	Other
152	880	29	16-32	Female	Urine	P.mirabilis
153	696	33	32-46	Female	Urine	Acinetobacter Spp
154	559	50	46-61	Female	Urine	E.coli
155	220	36	32-46	Female	Urine	E.coli
156	252	24	16-32	Female	Urine	E.coli
157	712	38	32-46	Female	Urine	K.pneumoniae
158	998	43	32-46	Female	Urine	E.coli
159	304	81	>=61	Male	Urine	E.coli
160	579	35	32-46	Male	Urine	E.coli
161	355	80	>=61	Male	Urine	Citrobacter Spp
162	353	32	16-32	Female	Urine	E.coli
163	217	32	16-32	Female	Other	K.pneumoniae
164	322	35	32-46	Male	Urine	E.coli
165	408	15	<=15	Male	Body fluid	K.pneumoniae
166	818	32	32-46	Female	Urine	E.coli
167	639	73	>=61	Male	Other	Acinetobacter Spp
168	498	50	46-61	Female	Wound	K.pneumoniae
169	715	57	46-61	Male	Urine	E.coli
170	149	6	<=15	Female	Urine	P.mirabilis
171	211	70	>=61	Male	Urine	E.coli
172	670	70	>=61	Male	Urine	E.coli
173	993	28	16-32	Male	Urine	Enterobacter Spp
174	173	22	16-32	Female	Urine	E.coli
175	509	13	<=15	Female	Wound	K.oxytoca & k.oz

	BetaLactamase	ESBLPhoenix	ESBL Manual	CTX	CRO	CAZ	FEP	ATM	CXM	XM	AMP	AMC
141	NEG	NEG	NEG	S	S	S	S	S	S	S	R	S
142	NEG	NEG	NEG	S	S	S	S	S	S	S	R	R
143	NEG	NEG	NEG	S	S	S	S	S	S	S	S	S
144	NEG	NEG	NEG	S	S	S	S	S	S	S	S	S
145	ESBL	POS	POS	R	R	R	R	R	R	R	R	S
146	NEG	NEG	NEG	S	S	S	S	S	S	S	R	R
147	ESBL	POS	POS	R	R	R	R	R	S	R	R	R
148	NEG	NEG	NEG	R	R	S	S	S	R	S	R	R
149	ESBL	POS	POS	R	R	R	R	R	R	R	R	R
150	NEG	NEG	NEG	S	S	S	S	S	R	S	R	R
151	NEG	NEG	NEG	S	S	S	S	S	R	S	R	R
152	NEG	NEG	NEG	R	R	S	R	R	R	R	R	R
153	NEG	NEG	NEG	S	S	S	S	S	S	S	R	R
154	NEG	NEG	NEG	S	S	S	S	S	S	S	R	R
155	NEG	NEG	NEG	S	S	S	S	S	S	S	S	S
156	ESBL	POS	POS	R	R	R	R	R	R	R	R	R
157	ESBL	POS	POS	R	R	R	R	R	R	R	R	R
158	ESBL	POS	POS	R	R	R	R	R	R	R	R	R
159	ESBL	POS	POS	R	R	R	R	R	R	R	R	R
160	ESBL	POS	POS	R	R	R	R	R	R	R	R	R
161	NEG	NEG	NEG	S	S	S	S	S	S	S	R	R
162	ESBL	POS	POS	R	R	R	R	R	R	R	R	R
163	NEG	NEG	NEG	S	S	S	S	S	S	S	R	R
164	NEG	NEG	NEG	S	S	S	S	S	S	S	R	S
165	NEG	NEG	NEG	R	R	R	R	R	R	R	R	R
166	ESBL	POS	POS	R	R	R	R	R	R	R	R	R
167	NEG	NEG	NEG	S	S	S	S	S	R	S	R	R
168	ESBL	POS	POS	R	R	R	R	R	R	R	R	R
169	NEG	NEG	NEG	S	S	S	S	S	R	S	R	S
170	NEG	NEG	NEG	S	S	S	S	S	R	S	R	S
171	NEG	NEG	NEG	S	S	S	S	S	S	S	S	S
172	NEG	NEG	NEG	S	S	S	S	S	S	S	R	R
173	NEG	NEG	NEG	R	R	S	R	R	R	R	R	R
174	NEG	NEG	NEG	S	S	S	S	S	S	S	R	R
175	NEG	NEG	NEG	S	S	S	S	S	S	S	R	S

	FOX	MEM	IMP	ETP	SXT	CIP	NOR	GM	AN	TZP	MDR2	MDR3	AmpC
141	S	S	S	S	S	S	S	S	S	S	R1	NO	NEG
142	S	S	S	S	R	R	S	S	S	S	R3	YES	NEG
143	S	S	S	S	S	S	S	S	S	S	RO	NO	NEG
144	S	S	S	S	S	S	S	S	S	S	RO	NO	NEG
145	R	S	S	S	R	R	R	S	S	S	R6	YES	POS
146	S	S	S	S	R	R	R	S	S	S	R4	YES	NEG
147	S	S	S	S	R	R	R	S	S	S	R6	YES	NEG
148	R	S	S	R	R	S	S	S	S	S	R7	YES	NEG
149	S	S	S	S	R	R	R	S	S	S	R7	YES	NEG
150	S	S	S	S	S	S	S	R	S	S	R3	YES	NEG
151	S	S	S	S	S	R	S	R	S	S	R3	YES	NEG
152	R	S	S	S	S	S	S	R	S	S	R6	YES	NEG
153	S	S	S	R	S	S	S	S	S	S	R3	YES	NEG
154	S	S	S	S	S	S	S	S	S	S	R2	NO	NEG
155	S	S	S	S	S	R	R	S	S	S	R1	NO	NEG
156	R	S	S	S	R	R	R	S	S	S	R8	YES	NEG
157	R	S	S	S	R	R	R	S	S	R	R9	YES	NEG
158	S	S	S	S	R	R	R	S	S	S	R7	YES	NEG
159	S	S	S	S	R	R	R	R	S	S	R7	YES	NEG
160	S	S	S	S	S	S	S	R	S	S	R5	YES	NEG
161	S	S	S	S	R	R	S	S	S	S	R4	YES	NEG
162	S	S	S	S	R	R	R	R	S	S	R8	YES	NEG
163	S	S	R	S	S	S	S	R	S	S	R3	YES	NEG
164	S	S	S	S	R	S	S	S	S	S	R2	NO	NEG
165	R	S	S	S	R	R	R	S	S	R	R9	YES	NEG
166	S	S	S	S	R	S	S	R	S	S	R7	YES	NEG
167	S	S	S	R	S	S	S	R	S	S	R5	YES	NEG
168	S	S	S	S	R	R	S	R	S	S	R6	YES	NEG
169	S	S	S	S	S	S	S	S	S	S	R2	NO	NEG
170	S	R	S	S	R	R	R	S	S	S	R4	YES	NEG
171	S	S	S	S	S	R	R	S	S	S	R1	NO	NEG
172	S	S	S	S	S	S	S	S	S	S	R2	NO	NEG
173	R	S	S	S	R	R	S	S	S	S	R7	YES	NEG
174	S	S	S	S	R	R	S	R	S	S	R3	YES	NEG
175	S	S	S	S	S	S	S	S	S	S	R1	NO	NEG

	BLCONFIRMATORY	GNBPOS	Healthcenter
141	NEG	POS	Private
142	NEG	POS	Governmen
143	NEG	POS	Governmen
144	NEG	POS	Private
145	POS	POS	Private
146	NEG	POS	Private
147	POS	POS	Governmen
148	NEG	POS	Private
149	POS	POS	Governmen
150	NEG	POS	Private
151	NEG	POS	Governmen
152	NEG	POS	Private
153	NEG	POS	Governmen
154	NEG	POS	Private
155	NEG	POS	Governmen
156	POS	POS	Private
157	POS	POS	Governmen
158	POS	POS	Private
159	POS	POS	Private
160	POS	POS	Governmen
161	NEG	POS	Governmen
162	POS	POS	Private
163	NEG	POS	Private
164	NEG	POS	Private
165	NEG	POS	Private
166	POS	POS	Private
167	NEG	POS	Private
168	POS	POS	Governmen
169	NEG	POS	Governmen
170	NEG	POS	Private
171	NEG	POS	Governmen
172	NEG	POS	Governmen
173	NEG	POS	Private
174	NEG	POS	Private
175	NEG	POS	Private

	ID	Age	AgeGroup	Sex	Specimen	GNB
176	804	13	<=15	Female	Wound	Other
177	483	65	>=61	Female	Urine	E.coli
178	947	23	16-32	Male	Urine	E.coli
179	958	23	16-32	Male	Urine	E.coli
180	447	38	32-46	Male	Wound	K.pneumoniae
181	448	50	46-61	Male	Wound	Enterobacter Spp
182	104	30	16-32	Male	Urine	E.coli
183	114	30	16-32	Female	Urine	E.coli
184	150	40	32-46	Female	Other	E.coli
185	186	84	>=61	Male	Urine	E.coli
186	171	57	46-61	Female	Urine	E.coli
187	165	57	46-61	Female	Urine	Other
188	175	55	46-61	Female	Urine	E.coli
189	900	23	16-32	Male	Urine	E.coli
190	205	82	>=61	Female	Urine	K.pneumoniae
191	390	22	16-32	Female	Urine	E.coli
192	200	26	16-32	Female	Urine	E.coli
193	603	53	46-61	Male	Wound	E.coli
194	881	24	16-32	Male	Urine	E.coli
195	480	75	>=61	Female	Urine	E.coli
196	819	45	32-46	Male	Wound	Enterobacter Spp
197	904	47	46-61	Male	Urine	E.coli
198	684	75	>=61	Female	Urine	E.coli
199	126	85	>=61	Female	Other	K.pneumoniae
200	226	85	>=61	Female	Other	Pseudomonas Spp
201	665	84	>=61	Female	Urine	E.coli
202	103	55	46-61	Female	Urine	E.coli
203	259	62	>=61	Male	Urine	E.coli
204	295	62	>=61	Male	Urine	E.coli
205	316	30	16-32	Female	Urine	E.coli
206	322	21	16-32	Male	Body fluid	E.coli
207	164	50	46-61	Male	Urine	E.coli
208	997	26	16-32	Female	Urine	E.coli
209	226	10	<=15	Female	Urine	K.pneumoniae
210	803	59	46-61	Female	Wound	E.coli

	BetaLactamase	ESBLPhoenix	ESBL Manual	CTX	CRO	CAZ	FEP	ATM	CXM	XM	AMP	AMC
176	NEG	NEG	NEG	S	S	S	S	S	S	S	R	S
177	ESBL	POS	POS	R	R	R	R	R	S	R	R	R
178	ESBL	POS	POS	S	S	S	S	S	S	S	R	R
179	NEG	NEG	NEG	S	S	S	S	S	S	S	S	R
180	ESBL	POS	POS	R	R	R	R	R	R	R	R	R
181	NEG	NEG	NEG	R	R	R	R	R	R	R	R	R
182	ESBL	POS	POS	R	R	R	R	R	R	R	R	R
183	NEG	NEG	NEG	S	S	S	S	S	R	S	S	S
184	NEG	NEG	NEG	S	S	S	S	S	S	S	S	S
185	ESBL	POS	POS	R	R	R	R	R	R	R	R	R
186	ESBL	POS	POS	R	R	R	R	R	S	R	R	R
187	NEG	NEG	NEG	R	S	S	S	R	S	R	R	R
188	ESBL	POS	POS	R	R	R	R	R	R	R	R	R
189	NEG	NEG	NEG	S	S	S	S	R	S	R	R	R
190	ESBL	POS	POS	R	R	R	R	R	R	R	R	R
191	NEG	NEG	NEG	S	S	S	S	S	S	S	S	S
192	ESBL	POS	POS	R	R	R	R	R	R	R	R	R
193	NEG	NEG	NEG	S	S	S	S	S	S	S	R	S
194	ESBL	POS	POS	R	R	R	R	R	R	R	R	R
195	ESBL	POS	POS	R	R	R	R	R	S	R	R	R
196	ESBL	POS	POS	R	R	R	R	R	R	R	R	R
197	ESBL	POS	POS	R	R	R	R	R	R	R	R	R
198	NEG	NEG	NEG	S	S	S	S	S	R	S	S	S
199	ESBL	POS	POS	R	R	R	R	R	R	R	R	R
200	NEG	NEG	NEG	R	R	S	S	S	R	S	R	R
201	NEG	NEG	NEG	S	S	S	S	S	R	S	R	R
202	NEG	NEG	NEG	S	S	S	S	S	R	S	R	R
203	ESBL	POS	POS	R	R	R	R	R	R	R	R	R
204	ESBL	POS	POS	R	R	R	R	R	S	R	R	R
205	NEG	NEG	NEG	S	S	S	S	S	S	S	S	S
206	NEG	NEG	NEG	S	S	S	S	S	S	S	S	R
207	ESBL	POS	POS	R	R	R	R	R	R	R	R	R
208	ESBL	POS	POS	R	R	R	R	R	R	R	R	R
209	NEG	NEG	NEG	S	S	S	S	S	S	S	R	S
210	NEG	NEG	NEG	S	S	S	S	S	S	S	R	S

	FOX	MEM	IMP	ETP	SXT	CIP	NOR	GM	AN	TZP	MDR2	MDR3	AmpC
176	S	S	S	S	S	S	S	S	S	S	R1	NO	NEG
177	R	S	S	S	R	S	S	S	S	S	R6	YES	NEG
178	S	S	S	S	R	S	S	R	S	S	R3	YES	NEG
179	S	S	S	S	S	S	S	S	S	S	R2	NO	NEG
180	R	R	S	R	R	R	R	R	S	R	R11	YES	NEG
181	R	S	S	S	R	S	S	R	S	S	R8	YES	NEG
182	S	S	S	S	S	R	R	S	S	S	R6	YES	NEG
183	S	S	S	S	S	R	R	S	S	S	R2	NO	NEG
184	S	S	S	S	S	R	R	S	S	S	R1	NO	NEG
185	S	S	S	S	R	R	S	S	S	S	R6	YES	NEG
186	S	S	S	S	R	R	S	S	S	S	R5	YES	NEG
187	S	S	R	S	R	S	S	S	S	S	R5	YES	NEG
188	S	S	S	S	R	S	S	S	S	S	R5	YES	NEG
189	R	S	S	S	S	S	S	S	S	S	R4	YES	NEG
190	S	S	S	S	R	R	S	S	S	S	R6	YES	NEG
191	S	S	S	S	S	S	S	S	S	S	RO	NO	NEG
192	S	S	S	S	R	S	S	S	S	S	R6	YES	NEG
193	S	S	S	S	R	S	S	S	S	S	R2	NO	NEG
194	R	S	S	S	R	S	S	S	S	S	R7	YES	NEG
195	S	S	S	S	R	R	R	S	S	S	R6	YES	NEG
196	R	S	S	S	R	R	R	R	S	S	R8	YES	NEG
197	R	S	S	S	R	R	S	S	S	S	R7	YES	NEG
198	S	S	S	S	S	S	S	S	S	S	R1	NO	NEG
199	S	S	S	S	R	R	R	S	S	S	R7	YES	NEG
200	R	S	S	R	R	S	S	S	S	S	R7	YES	NEG
201	S	S	S	S	S	S	S	S	S	S	R3	YES	NEG
202	S	S	S	S	S	S	S	S	S	S	R3	YES	NEG
203	S	S	S	S	R	S	S	S	S	S	R6	YES	NEG
204	S	S	S	S	R	S	S	S	S	S	R5	YES	NEG
205	S	S	S	S	S	S	S	S	S	S	RO	NO	NEG
206	S	S	S	S	R	S	S	S	S	S	R8	YES	NEG
207	S	S	S	S	R	R	R	R	S	S	R3	YES	NEG
208	R	S	S	S	R	S	S	S	S	R	R8	YES	NEG
209	S	S	S	S	S	R	R	S	S	S	R2	NO	NEG
210	S	S	S	S	S	R	R	S	S	S	R2	NO	NEG

	BLCONFIRMATORY	GNBPOS	Healthcenter
176	NEG	POS	Private
177	NEG	POS	Private
178	POS	POS	Governmen
179	NEG	POS	Governmen
180	POS	POS	Private
181	NEG	POS	Private
182	POS	POS	Governmen
183	NEG	POS	Private
184	NEG	POS	Governmen
185	POS	POS	Private
186	POS	POS	Governmen
187	NEG	POS	Private
188	POS	POS	Governmen
189	NEG	POS	Private
190	POS	POS	Governmen
191	NEG	POS	Private
192	POS	POS	Private
193	NEG	POS	Private
194	POS	POS	Governmen
195	POS	POS	Governmen
196	POS	POS	Private
197	POS	POS	Private
198	NEG	POS	Private
199	POS	POS	Governmen
200	NEG	POS	Private
201	NEG	POS	Private
202	NEG	POS	Private
203	POS	POS	Private
204	POS	POS	Governmen
205	NEG	POS	Private
206	NEG	POS	Governmen
207	POS	POS	Private
208	POS	POS	Governmen
209	NEG	POS	Private
210	NEG	POS	Private

	ID	Age	AgeGroup	Sex	Specimen	GNB
211	802	59	46-61	Female	Wound	E.coli
212	878	39	32-46	Male	Wound	K.pneumoniae
213	130	64	>=61	Male	Urine	E.coli
214	136	64	>=61	Male	Urine	E.coli
215	257	42	32-46	Male	Urine	Citrobacter Spp
216	275	42	32-46	Male	Urine	Citrobacter Spp
217	600	3	<=15	Female	Urine	E.coli
218	523	53	46-61	Female	Urine	E.coli
219	553	53	46-61	Female	Urine	E.coli
220	850	58	46-61	Male	Urine	Citrobacter Spp
221	385	58	46-61	Male	Urine	K.pneumoniae
222	332	42	32-46	Female	Urine	E.coli
223	215	59	46-61	Female	Urine	E.coli
224	975	62	>=61	Female	Urine	E.coli
225	402	24	16-32	Male	Wound	E.coli
226	337	50	46-61	Male	Urine	E.coli
227	137	35	32-46	Male	Wound	Enterobacter Spp
228	602	29	16-32	Female	Urine	E.coli
229	580	65	>=61	Female	Urine	K.pneumoniae
230	382	21	16-32	Male	Urine	E.coli
231	170	40	32-46	Male	Wound	E.coli
232	237	9	<=15	Female	Urine	E.coli
233	464	3	<=15	Female	Urine	E.coli
234	463	31	16-32	Female	Urine	E.coli
235	570	53	46-61	Female	Urine	Pseudomonas Spp
236	558	25	16-32	Female	Urine	E.coli
237	590	25	16-32	Female	Urine	E.coli
238	645	50	46-61	Female	Wound	Pseudomonas Spp
239	664	50	46-61	Female	Wound	K.pneumoniae
240	260	27	16-32	Female	Urine	E.coli
241	637	29	16-32	Female	Urine	E.coli
242	654	16	16-32	Female	Other	K.oxytoca & k.oz
243	172	38	32-46	Female	Urine	E.coli
244	633	70	>=61	Male	Urine	E.coli
245	208	46	46-61	Female	Other	E.coli

	BetaLactamase	ESBLPhoenix	ESBL Manual	CTX	CRO	CAZ	FEP	ATM	CXM	XM	AMP	AMC
211	NEG	NEG	NEG	S	S	S	S	S	S	S	R	S
212	ESBL	POS	POS	R	R	R	R	R	S	R	R	R
213	NEG	NEG	NEG	R	R	S	R	R	S	R	R	R
214	ESBL	POS	POS	R	R	R	R	R	R	R	R	R
215	NEG	NEG	NEG	S	S	S	S	R	S	R	R	R
216	NEG	NEG	NEG	S	S	S	S	R	S	R	R	R
217	NEG	NEG	NEG	S	S	S	S	S	S	S	R	S
218	ESBL	POS	POS	R	R	R	R	R	R	R	R	R
219	ESBL	POS	POS	R	R	R	R	R	R	R	R	R
220	NEG	NEG	NEG	S	S	S	S	R	S	R	R	R
221	NEG	NEG	NEG	R	R	S	R	R	R	R	R	S
222	ESBL	POS	POS	R	R	R	R	R	R	R	R	R
223	NEG	NEG	NEG	S	S	S	S	S	S	S	R	S
224	NEG	NEG	NEG	S	S	S	S	S	S	S	R	R
225	NEG	NEG	NEG	S	S	S	S	S	S	S	S	S
226	ESBL	POS	POS	R	R	R	R	R	R	R	R	R
227	ESBL	POS	POS	R	R	R	R	R	S	R	R	R
228	NEG	NEG	NEG	R	S	S	S	S	S	S	R	R
229	ESBL	POS	POS	R	R	R	R	R	R	R	R	S
230	NEG	NEG	NEG	R	S	S	S	R	S	R	S	S
231	NEG	NEG	NEG	S	S	S	S	S	S	S	R	S
232	NEG	NEG	NEG	S	S	S	S	S	S	S	R	S
233	ESBL	POS	POS	R	R	R	R	R	R	R	R	R
234	ESBL	POS	NEG	R	R	R	R	R	R	R	R	R
235	NEG	NEG	NEG	S	S	S	S	S	S	S	R	R
236	NEG	NEG	NEG	S	S	S	S	S	S	S	S	S
237	ESBL	POS	NEG	R	R	R	R	R	R	R	R	R
238	NEG	NEG	NEG	R	R	S	S	S	S	S	R	R
239	NEG	NEG	NEG	S	S	S	S	S	R	S	R	R
240	ESBL	POS	POS	R	R	R	R	R	R	R	R	R
241	NEG	NEG	NEG	S	S	S	S	S	S	S	R	R
242	NEG	NEG	NEG	S	S	S	S	S	S	S	R	S
243	NEG	NEG	NEG	S	S	S	S	S	S	S	R	S
244	NEG	NEG	NEG	S	S	S	S	S	S	S	S	S
245	NEG	NEG	NEG	S	S	S	S	S	S	S	S	S

	FOX	MEM	IMP	ETP	SXT	CIP	NOR	GM	AN	TZP	MDR2	MDR3	AmpC
211	S	S	S	S	S	R	R	S	S	S	R2	NO	NEG
212	S	S	S	S	R	R	R	R	S	R	R8	YES	NEG
213	R	S	S	S	R	R	R	S	S	R	R8	YES	POS
214	S	S	S	S	S	S	S	S	S	S	R5	YES	NEG
215	R	S	R	S	R	S	S	S	S	S	R6	YES	POS
216	S	S	R	S	R	R	R	R	S	S	R6	YES	NEG
217	S	S	S	S	R	S	S	S	S	S	R2	NO	NEG
218	S	S	S	S	S	S	S	S	S	S	R5	YES	NEG
219	S	S	S	S	S	S	S	S	S	S	R5	YES	NEG
220	R	S	S	S	S	S	S	S	S	R	R5	YES	NEG
221	R	S	S	R	S	S	S	S	S	R	R7	YES	NEG
222	S	S	S	S	R	S	S	S	S	S	R6	YES	NEG
223	S	S	S	S	S	S	S	S	S	S	R1	NO	NEG
224	S	S	S	S	R	R	S	S	S	S	R3	YES	NEG
225	S	S	S	S	R	S	S	S	S	S	R1	NO	NEG
226	S	S	S	S	R	R	R	R	S	S	R7	YES	NEG
227	R	S	S	S	R	R	R	R	S	R	R9	YES	NEG
228	S	S	S	S	R	R	S	S	S	S	R3	YES	NEG
229	S	S	S	S	R	S	S	S	S	S	R5	YES	NEG
230	S	S	S	S	R	S	S	S	S	S	R2	NO	NEG
231	R	S	S	S	R	R	S	R	R	S	R4	YES	NEG
232	S	S	S	S	R	S	S	S	S	S	R2	NO	NEG
233	S	S	S	S	S	S	S	S	S	S	R5	YES	NEG
234	S	S	S	S	S	R	R	S	S	S	R6	YES	NEG
235	S	S	S	R	S	S	S	R	S	S	R3	YES	NEG
236	S	S	S	S	S	S	S	S	S	S	RO	NO	NEG
237	R	S	S	S	R	R	R	R	S	S	R9	YES	NEG
238	R	S	S	R	R	S	S	S	S	S	R6	YES	NEG
239	S	S	S	S	R	S	S	S	S	S	R4	YES	NEG
240	S	S	S	S	R	R	R	S	S	S	R7	YES	NEG
241	S	S	S	S	R	R	R	S	S	S	R4	YES	NEG
242	S	S	S	S	S	S	S	S	S	S	R1	NO	NEG
243	S	S	S	S	R	S	S	S	S	S	R2	NO	NEG
244	S	S	S	S	S	S	S	S	S	S	RO	NO	NEG
245	S	S	S	S	S	S	S	S	S	S	RO	NO	NEG

	BLCONFIRMATORY	GNBPOS	Healthcenter
211	NEG	POS	Private
212	POS	POS	Governmen
213	NEG	POS	Governmen
214	POS	POS	Private
215	POS	POS	Governmen
216	NEG	POS	Private
217	NEG	POS	Private
218	POS	POS	Private
219	POS	POS	Private
220	POS	POS	Governmen
221	NEG	POS	Governmen
222	POS	POS	Private
223	NEG	POS	Governmen
224	NEG	POS	Private
225	NEG	POS	Private
226	POS	POS	Governmen
227	POS	POS	Private
228	NEG	POS	Governmen
229	POS	POS	Private
230	NEG	POS	Governmen
231	NEG	POS	Governmen
232	NEG	POS	Private
233	POS	POS	Private
234	NEG	POS	Governmen
235	NEG	POS	Private
236	NEG	POS	Governmen
237	POS	POS	Private
238	NEG	POS	Governmen
239	NEG	POS	Governmen
240	POS	POS	Private
241	NEG	POS	Private
242	NEG	POS	Governmen
243	NEG	POS	Governmen
244	NEG	POS	Private
245	NEG	POS	Private

	ID	Age	AgeGroup	Sex	Specimen	GNB
246	685	26	16-32	Female	Urine	E.coli
247	992	21	16-32	Female	Urine	E.coli
248	771	35	32-46	Female	Wound	E.coli
249	659	32	32-46	Male	Wound	Pseudomonas Spp
250	559	46	46-61	Female	Wound	K.pneumoniae
251	696	1	<=15	Female	Urine	K.pneumoniae
252	680	71	>=61	Male	Urine	E.coli
253	672	80	>=61	Female	Urine	Citrobacter Spp
254	667	29	16-32	Female	Urine	E.coli
255	240	45	32-46	Female	Urine	E.coli
256	293	29	16-32	Male	Urine	Other
257	219	29	16-32	Female	Urine	E.coli
258	705	9	<=15	Male	Other	Pseudomonas Spp
259	613	84	>=61	Male	Urine	E.coli
260	479	23	16-32	Female	Urine	E.coli
261	431	65	>=61	Female	Urine	E.coli
262	499	65	>=61	Female	Urine	E.coli
263	595	65	>=61	Female	Urine	E.coli
264	284	62	>=61	Female	Urine	E.coli
265	300	24	16-32	Male	Other	E.coli
266	112	70	>=61	Female	Urine	E.coli
267	388	64	>=61	Male	Urine	E.coli
268	829	45	32-46	Female	Urine	E.coli
269	549	36	32-46	Female	Urine	E.coli
270	239	8	<=15	Female	Urine	E.coli
271	555	40	32-46	Male	Wound	E.coli
272	765	40	32-46	Male	Wound	Citrobacter Spp
273	127	18	16-32	Male	Wound	E.coli
274	275	19	16-32	Female	Urine	K.pneumoniae
275	135	57	46-61	Female	Urine	E.coli
276	306	47	46-61	Male	Wound	Pseudomonas Spp
277	326	56	46-61	Female	Urine	E.coli
278	124	44	32-46	Male	Urine	E.coli
279	713	25	16-32	Male	Urine	K.pneumoniae
280	344	27	16-32	Male	Urine	E.coli

	BetaLactamase	ESBLPhoenix	ESBL Manual	CTX	CRO	CAZ	FEP	ATM	CXM	XM	AMP	AMC
246	NEG	NEG	NEG	S	S	S	S	S	S	S	R	R
247	NEG	NEG	NEG	S	S	S	S	S	S	S	R	R
248	NEG	NEG	NEG	S	S	S	S	S	S	S	R	R
249	NEG	NEG	NEG	R	R	S	S	S	R	S	R	R
250	NEG	NEG	NEG	S	S	S	S	S	S	S	R	R
251	NEG	NEG	NEG	R	R	R	S	R	R	R	R	S
252	NEG	NEG	NEG	S	S	S	S	S	S	S	S	S
253	ESBL	POS	POS	R	R	R	R	R	S	R	R	R
254	NEG	NEG	NEG	S	S	S	S	S	S	S	R	R
255	NEG	NEG	NEG	S	S	S	S	S	R	S	R	R
256	NEG	NEG	NEG	S	S	S	S	S	S	S	R	R
257	ESBL	POS	POS	R	R	R	R	R	S	R	R	R
258	NEG	NEG	NEG	R	R	S	S	S	R	S	R	R
259	ESBL	POS	POS	R	R	R	R	R	R	R	R	R
260	ESBL	POS	POS	R	R	R	R	R	R	R	R	R
261	NEG	NEG	NEG	S	S	S	S	S	R	S	S	S
262	ESBL	POS	POS	R	R	R	R	R	R	R	R	R
263	ESBL	POS	POS	R	R	R	R	R	R	R	R	R
264	NEG	NEG	NEG	S	S	S	S	S	S	S	R	R
265	NEG	NEG	NEG	R	R	R	R	R	R	R	R	R
266	ESBL	POS	POS	R	R	R	R	R	R	R	R	R
267	ESBL	POS	POS	R	R	R	R	R	R	R	R	R
268	ESBL	POS	POS	R	R	R	R	R	R	R	R	R
269	ESBL	POS	POS	R	R	R	R	R	R	R	R	R
270	NEG	NEG	NEG	S	S	S	S	S	R	S	R	S
271	ESBL	POS	NEG	R	R	R	R	R	R	R	R	R
272	NEG	NEG	NEG	S	S	S	S	S	R	S	R	R
273	ESBL	POS	POS	R	R	R	R	R	S	R	R	R
274	NEG	NEG	NEG	S	S	S	S	S	R	S	R	R
275	NEG	NEG	NEG	R	R	R	R	R	R	R	R	R
276	NEG	NEG	NEG	R	R	S	S	S	R	S	R	R
277	NEG	NEG	NEG	S	S	S	S	S	S	S	S	S
278	ESBL	POS	POS	R	R	R	R	R	R	R	R	R
279	ESBL	POS	POS	R	R	R	R	R	R	R	R	R
280	ESBL	POS	POS	R	R	R	R	R	S	R	R	R

	FOX	MEM	IMP	ETP	SXT	CIP	NOR	GM	AN	TZP	MDR2	MDR3	AmpC
246	S	S	S	S	R	S	S	S	S	S	R3	YES	NEG
247	S	S	S	S	R	S	S	S	S	S	R3	YES	NEG
248	S	S	S	S	R	R	R	S	S	S	R4	YES	NEG
249	R	S	S	R	R	R	S	S	S	S	R7	YES	NEG
250	S	R	R	R	S	S	S	S	S	S	R3	YES	NEG
251	R	S	S	S	R	R	R	S	S	S	R7	YES	NEG
252	S	S	S	S	S	S	S	S	S	S	RO	NO	NEG
253	S	S	S	S	R	R	R	S	S	S	R3	YES	NEG
254	S	S	S	S	R	S	S	S	S	S	R3	YES	NEG
255	S	S	S	S	R	R	R	S	S	S	R5	YES	NEG
256	R	S	S	S	S	S	S	S	S	S	R3	YES	NEG
257	S	S	S	S	R	R	R	S	S	S	R6	YES	NEG
258	S	S	S	R	R	S	S	S	S	S	R6	YES	NEG
259	S	S	S	S	R	R	R	S	S	S	R7	YES	NEG
260	S	S	S	S	R	R	R	S	S	S	R7	YES	NEG
261	S	S	S	S	S	S	S	S	S	S	R1	NO	NEG
262	S	S	S	S	R	R	R	R	S	S	R8	YES	NEG
263	S	S	S	S	R	S	R	R	S	S	R8	YES	NEG
264	S	S	S	S	S	R	S	S	S	S	R3	YES	NEG
265	R	S	S	S	R	R	R	S	S	R	R9	YES	NEG
266	S	S	S	S	R	R	R	S	S	S	R7	YES	NEG
267	S	S	S	S	S	R	R	R	S	S	R7	YES	NEG
268	S	S	S	S	S	S	S	S	S	S	R5	YES	NEG
269	S	S	S	S	S	R	R	R	S	S	R7	YES	NEG
270	S	S	S	S	S	S	S	R	S	S	R3	YES	NEG
271	R	S	S	S	R	R	R	S	S	R	R9	YES	NEG
272	R	S	S	S	S	S	S	S	S	S	R4	YES	NEG
273	S	S	S	S	R	R	R	S	S	R	R7	YES	NEG
274	S	S	S	S	R	S	S	S	S	S	R4	YES	NEG
275	S	S	S	R	S	R	R	S	S	R	R9	YES	NEG
276	R	S	S	R	R	S	S	S	S	S	R7	YES	NEG
277	S	S	S	S	S	S	S	S	S	S	RO	NO	NEG
278	R	S	S	S	R	R	R	S	S	S	R7	YES	NEG
279	S	S	S	S	R	R	R	S	S	S	R7	YES	NEG
280	S	S	S	S	R	S	S	S	S	S	R5	YES	NEG

	BLCONFIRMATORY	GNBPOS	Healthcenter
246	NEG	POS	Private
247	NEG	POS	Governmen
248	NEG	POS	Private
249	NEG	POS	Governmen
250	NEG	POS	Private
251	NEG	POS	Private
252	NEG	POS	Governmen
253	POS	POS	Governmen
254	NEG	POS	Governmen
255	NEG	POS	Private
256	NEG	POS	Private
257	POS	POS	Private
258	NEG	POS	Governmen
259	POS	POS	Governmen
260	POS	POS	Private
261	NEG	POS	Private
262	POS	POS	Governmen
263	POS	POS	Governmen
264	NEG	POS	Private
265	NEG	POS	Governmen
266	POS	POS	Private
267	POS	POS	Private
268	POS	POS	Private
269	POS	POS	Private
270	NEG	POS	Private
271	NEG	POS	Governmen
272	NEG	POS	Governmen
273	POS	POS	Governmen
274	NEG	POS	Governmen
275	POS	POS	Private
276	NEG	POS	Governmen
277	NEG	POS	Private
278	POS	POS	Governmen
279	POS	POS	Private
280	POS	POS	Governmen

	ID	Age	AgeGroup	Sex	Specimen	GNB
281	284	62	>=61	Female	Urine	E.coli
282	458	15	<=15	Male	Wound	E.coli
283	856	4	<=15	Female	Urine	E.coli
284	644	40	32-46	Male	Urine	K.pneumoniae
285	789	40	32-46	Female	Wound	Other
286	441	73	>=61	Male	Urine	E.coli
287	168	58	46-61	Female	Urine	E.coli
288	280	75	>=61	Female	Urine	E.coli
289	407	48	46-61	Male	Wound	Other
290	585	72	>=61	Female	Wound	E.coli
291	356	62	>=61	Female	Wound	K.pneumoniae
292	379	62	>=61	Female	Wound	Pseudomonas Spp
293	671	4	<=15	Male	Urine	E.coli
294	601	83	>=61	Female	Urine	E.coli
295	899	28	16-32	Female	Urine	E.coli
296	687	70	>=61	Male	Wound	E.coli
297	678	47	46-61	Female	Urine	E.coli
298	619	22	16-32	Female	Urine	Enterobacter Spp
299	710	62	>=61	Male	Urine	E.coli
300	560	65	>=61	Male	Urine	E.coli
301	752	60	>=61	Male	Urine	E.coli
302	727	28	16-32	Male	Other	Shigella Spp
303	698	57	46-61	Female	Urine	E.coli
304	794	41	32-46	Male	Wound	E.coli
305	792	3	<=15	Female	Urine	E.coli
306	915	75	>=61	Male	Urine	E.coli
307	382	20	16-32	Female	Urine	E.coli
308	192	23	16-32	Female	Urine	E.coli
309	666	76	>=61	Male	Wound	K.pneumoniae
310	550	76	>=61	Male	Wound	Pseudomonas Spp
311	318	5	<=15	Female	Urine	E.coli
312	194	41	32-46	Male	Urine	E.coli
313	405	60	46-61	Female	Urine	K.pneumoniae
314	399	55	>=61	Female	Urine	E.coli
315	779	62	>=61	Female	Urine	E.coli

	BetaLactamase	ESBLPhoenix	ESBL Manual	CTX	CRO	CAZ	FEP	ATM	CXM	XM	AMP	AMC
281	NEG	NEG	NEG	S	S	S	S	S	R	S	R	S
282	ESBL	POS	POS	R	R	R	R	R	R	R	R	R
283	NEG	NEG	NEG	S	S	S	S	S	R	S	R	S
284	ESBL	POS	POS	R	R	R	R	R	S	R	R	R
285	NEG	NEG	NEG	R	R	S	S	S	R	S	R	R
286	NEG	NEG	NEG	S	S	S	S	S	S	S	S	S
287	NEG	NEG	NEG	S	S	S	S	S	R	S	R	R
288	ESBL	POS	POS	R	R	R	R	R	R	R	R	R
289	NEG	NEG	NEG	S	S	S	S	S	S	S	R	R
290	ESBL	POS	POS	R	R	R	R	R	S	R	R	R
291	ESBL	POS	POS	R	R	R	R	R	R	R	R	R
292	NEG	NEG	NEG	R	R	R	R	R	R	R	R	R
293	NEG	NEG	NEG	S	S	S	R	R	R	R	S	S
294	ESBL	POS	POS	R	R	R	R	R	R	R	R	R
295	ESBL	POS	POS	R	R	R	R	R	R	R	R	R
296	ESBL	POS	POS	R	R	R	R	R	S	R	R	R
297	NEG	NEG	NEG	S	S	S	S	S	R	S	R	R
298	NEG	NEG	NEG	S	S	S	S	S	R	S	R	R
299	ESBL	POS	POS	R	R	R	R	R	R	R	R	R
300	ESBL	POS	POS	R	R	R	R	R	S	R	R	R
301	ESBL	POS	POS	R	R	R	R	R	R	R	R	R
302	NEG	NEG	NEG	S	S	S	S	S	R	S	R	S
303	ESBL	POS	POS	R	R	R	R	R	R	R	R	R
304	ESBL	POS	POS	R	R	R	R	R	R	R	R	R
305	NEG	NEG	NEG	S	S	S	S	S	R	S	S	S
306	ESBL	POS	POS	R	R	R	R	R	R	R	R	R
307	NEG	NEG	NEG	S	S	S	S	S	R	S	R	S
308	NEG	NEG	NEG	S	S	S	S	S	S	S	S	S
309	ESBL	POS	POS	R	R	R	R	R	R	R	R	R
310	NEG	NEG	NEG	R	R	R	R	R	S	R	R	R
311	NEG	NEG	NEG	S	S	S	S	S	S	S	S	S
312	ESBL	POS	POS	R	R	R	R	R	R	R	R	R
313	NEG	NEG	NEG	S	S	S	S	S	R	S	R	S
314	ESBL	POS	POS	R	R	R	R	R	S	R	R	R
315	ESBL	POS	POS	R	R	R	R	R	R	R	R	R

	FOX	MEM	IMP	ETP	SXT	CIP	NOR	GM	AN	TZP	MDR2	MDR3	AmpC
281	S	S	S	S	R	S	S	S	S	S	R3	YES	NEG
282	S	S	S	S	S	S	S	S	S	S	R5	YES	NEG
283	S	S	S	S	R	S	S	S	S	S	R3	YES	NEG
284	S	S	S	S	R	R	R	S	S	S	R6	YES	NEG
285	R	S	S	R	S	S	S	S	S	R	R7	YES	NEG
286	S	S	S	S	S	S	S	S	S	S	RO	NO	NEG
287	S	S	S	S	R	S	S	S	S	S	R4	YES	NEG
288	S	S	S	S	R	R	R	R	S	S	R7	YES	NEG
289	R	S	S	R	S	R	R	S	S	S	R5	YES	NEG
290	S	S	S	S	R	R	R	S	S	S	R6	YES	NEG
291	S	S	S	S	R	S	S	S	S	S	R6	YES	NEG
292	R	S	S	R	R	S	S	S	S	R	R9	YES	NEG
293	S	R	S	R	S	S	S	S	S	S	R4	YES	NEG
294	S	S	S	S	R	R	R	R	S	S	R7	YES	NEG
295	S	S	S	S	S	R	R	R	S	S	R7	YES	NEG
296	S	S	S	S	R	R	R	S	S	S	R6	YES	NEG
297	S	S	S	S	R	S	S	S	S	S	R4	YES	NEG
298	R	S	S	S	S	S	S	S	S	S	R4	YES	NEG
299	S	S	S	S	R	R	R	S	S	S	R7	YES	NEG
300	R	S	S	R	R	R	R	S	S	R	R9	YES	NEG
301	S	S	S	S	R	S	S	S	S	S	R6	YES	NEG
302	R	S	S	S	R	S	S	R	R	S	R5	YES	NEG
303	S	S	S	S	R	R	R	R	S	R	R9	YES	NEG
304	S	S	S	S	R	R	R	R	S	S	R8	YES	NEG
305	S	S	S	S	S	S	S	S	S	S	R1	NO	NEG
306	S	S	S	S	S	R	R	S	S	R	R7	YES	NEG
307	S	S	S	S	R	S	S	R	S	S	R4	YES	NEG
308	S	S	S	S	S	S	S	S	S	S	RO	NO	NEG
309	R	R	R	S	R	S	S	R	S	R	R10	YES	NEG
310	R	S	S	R	R	S	S	S	S	R	R8	YES	NEG
311	S	S	S	S	S	S	S	S	S	S	RO	NO	NEG
312	S	S	S	S	R	R	R	R	S	R	R9	YES	NEG
313	S	S	S	S	R	S	S	S	S	S	R3	YES	NEG
314	S	S	S	S	R	S	S	S	S	S	R5	YES	NEG
315	S	S	S	S	R	R	R	S	S	S	R7	YES	NEG

	BLCONFIRMATORY	GNBPOS	Healthcenter
281	NEG	POS	Governmen
282	POS	POS	Private
283	NEG	POS	Private
284	POS	POS	Private
285	NEG	POS	Governmen
286	NEG	POS	Private
287	NEG	POS	Governmen
288	POS	POS	Private
289	NEG	POS	Governmen
290	POS	POS	Governmen
291	POS	POS	Private
292	NEG	POS	Governmen
293	NEG	POS	Private
294	POS	POS	Private
295	POS	POS	Governmen
296	POS	POS	Private
297	NEG	POS	Governmen
298	NEG	POS	Private
299	POS	POS	Governmen
300	POS	POS	Private
301	POS	POS	Private
302	NEG	POS	Governmen
303	POS	POS	Private
304	POS	POS	Governmen
305	NEG	POS	Private
306	POS	POS	Governmen
307	NEG	POS	Private
308	NEG	POS	Governmen
309	POS	POS	Private
310	NEG	POS	Private
311	NEG	POS	Private
312	POS	POS	Governmen
313	NEG	POS	Private
314	POS	POS	Governmen
315	POS	POS	Private

	ID	Age	AgeGroup	Sex	Specimen	GNB
316	928	60	46-61	Female	Wound	Citrobacter Spp
317	920	80	>=61	Female	Urine	E.coli
318	923	33	32-46	Male	Urine	E.coli
319	131	30	16-32	Female	Urine	E.coli
320	263	51	46-61	Male	Urine	E.coli
321	134	44	32-46	Male	Urine	E.coli
322	177	34	16-32	Female	Urine	E.coli
323	148	40	32-46	Female	Urine	E.coli
324	962	62	>=61	Female	Urine	E.coli
325	249	34	32-46	Female	Urine	E.coli
326	142	37	32-46	Female	Urine	E.coli
327	943	29	32-46	Female	Urine	E.coli
328	946	1	<=15	Female	Urine	E.coli
329	140	55	46-61	Male	Wound	Other
330	190	31	16-32	Male	Wound	E.coli
331	232	67	>=61	Male	Urine	E.coli
332	121	27	16-32	Male	Urine	E.coli
333	369	21	16-32	Male	Other	P.mirabilis
334	122	24	16-32	Female	Wound	E.coli
335	167	70	>=61	Female	Urine	E.coli
336	162	60	46-61	Male	Urine	E.coli
337	181	43	32-46	Female	Urine	Pseudomonas Spp
338	171	38	32-46	Male	Urine	E.coli

	BetaLactamase	ESBLPhoenix	ESBL Manual	CTX	CRO	CAZ	FEP	ATM	CXM	XM	AMP	AMC
316	NEG	NEG	NEG	S	S	S	S	S	S	S	R	R
317	NEG	NEG	NEG	S	S	S	S	S	R	S	S	S
318	NEG	NEG	NEG	S	S	S	S	S	R	S	R	R
319	NEG	NEG	NEG	S	S	S	S	S	R	S	R	R
320	NEG	NEG	NEG	S	S	S	S	S	S	S	S	S
321	NEG	NEG	NEG	S	S	S	S	S	S	S	R	R
322	NEG	NEG	NEG	S	S	S	S	S	R	S	S	S
323	NEG	NEG	NEG	R	R	R	R	R	S	R	R	R
324	ESBL	POS	POS	R	R	R	R	R	S	R	R	R
325	NEG	NEG	NEG	S	S	S	S	S	S	S	R	R
326	NEG	NEG	NEG	S	S	S	S	S	S	S	S	S
327	NEG	NEG	NEG	S	S	S	S	S	S	S	R	S
328	NEG	NEG	NEG	S	S	S	S	S	S	S	S	S
329	NEG	NEG	NEG	S	S	S	S	S	S	S	R	R
330	NEG	NEG	NEG	S	S	S	S	S	S	S	R	S
331	ESBL	POS	POS	R	R	R	R	R	R	R	R	R
332	NEG	NEG	NEG	S	S	S	S	S	S	S	R	R
333	NEG	NEG	NEG	S	S	S	S	S	S	S	S	S
334	NEG	NEG	NEG	S	S	S	S	S	S	S	S	S
335	ESBL	POS	POS	S	R	R	R	R	R	R	R	R
336	ESBL	POS	POS	R	R	R	R	R	R	R	R	R
337	NEG	NEG	NEG	R	R	R	R	R	R	R	R	R
338	NEG	NEG	NEG	R	R	R	R	R	R	R	R	R

Row Data.sav

	FOX	MEM	IMP	ETP	SXT	CIP	NOR	GM	AN	TZP	MDR2	MDR3	AmpC
316	R	S	S	S	S	S	S	S	S	S	R3	YES	NEG
317	S	S	S	S	S	S	S	S	S	S	R1	NO	NEG
318	S	S	S	S	S	S	S	S	S	S	R3	YES	NEG
319	S	S	S	S	R	S	S	S	S	S	R4	YES	NEG
320	S	S	S	S	S	S	S	S	S	S	RO	NO	NEG
321	S	S	S	S	R	S	S	S	S	S	R3	YES	NEG
322	S	S	S	S	S	S	S	S	S	S	R1	NO	NEG
323	R	S	S	S	R	S	S	S	S	S	R6	YES	NEG
324	S	S	S	S	R	S	S	S	S	S	R5	YES	NEG
325	S	S	S	S	R	S	S	S	S	S	R3	YES	NEG
326	S	S	S	S	R	S	S	S	S	S	R1	NO	NEG
327	S	S	S	S	R	S	S	S	S	S	R2	NO	NEG
328	S	S	S	S	R	S	S	S	S	S	R1	NO	NEG
329	R	S	S	S	S	S	S	S	S	S	R3	YES	NEG
330	S	S	S	S	R	R	R	S	S	S	R3	YES	NEG
331	S	S	S	S	R	S	S	S	S	S	R6	YES	NEG
332	S	S	S	S	R	S	S	S	S	S	R3	YES	NEG
333	S	S	S	S	S	S	S	S	S	S	RO	NO	NEG
334	S	S	S	S	S	S	S	S	S	S	RO	NO	NEG
335	R	S	S	S	S	S	S	S	S	S	R4	YES	NEG
336	S	S	S	S	S	S	S	S	S	S	R5	YES	NEG
337	R	S	S	R	R	S	S	S	S	R	R9	YES	NEG
338	R	S	S	S	R	S	S	S	S	R	R8	YES	NEG

	BLCONFIR MATORY	GNBPOS	Healthcenter
316	NEG	POS	Private
317	NEG	POS	Governmen
318	NEG	POS	Private
319	NEG	POS	Governmen
320	NEG	POS	Private
321	NEG	POS	Governmen
322	NEG	POS	Governmen
323	NEG	POS	Governmen
324	POS	POS	Private
325	NEG	POS	Governmen
326	NEG	POS	Private
327	NEG	POS	Governmen
328	NEG	POS	Private
329	NEG	POS	Governmen
330	NEG	POS	Private
331	POS	POS	Governmen
332	NEG	POS	Governmen
333	NEG	POS	Governmen
334	NEG	POS	Private
335	POS	POS	Governmen
336	POS	POS	Private
337	NEG	POS	Governmen
338	NEG	POS	Private
