# Supplementary material for: Extended-spectrum Beta-lactamase and AmpC beta-lactamases producing gram negative bacilli isolated from clinical specimens at International Clinical Laboratories, Addis Ababa, Ethiopia
Source: PLoS One. 2020 Nov 12;15(11):e0241984. doi: 10.1371/journal.pone.0241984 (PMC7660541; doi:10.1371/journal.pone.0241984)
Supplement: S2 Data — (DOCX) [file pone.0241984.s002.docx]

## Data collection form for gram negative isolates

1. Isolate identification number:________________________
2. Age _______ Sex ______
3. Identification number of the isolate for the study:_____________________
4. Type of specimen:__________________________
5. Health facilities : A. Government B. Private
6. Isolated gram negative bacteria:
7. *Escherichia coli*
8. *Klebsiella pneumoniae,*
9. *Klebsiella oxytoca*
10. *Proteus mirabilis*
11. *Proteus vulgaris*
12. *Enterobacter cloacae*
13. *Enterobacter aerogenes*
14. *Citrobacter freundii*
15. *Serratia*
16. *Providencia*
17. *Salmonella*
18. *Shigella*
19. *Pseudomonas aeroginosa*
20. *Acinetobacter Baumanii*
21. *Other-------------*
22. Result of AST pattern of the isolates at ICL:

| S.No | Antimicrobial Agent | MIC | | | Result of AST (S/I/R)/(MIC) | Comments |
| --- | --- | --- | --- | --- | --- | --- |
|  |  | S | I | R |  |  |
|  | Ampicilin | ≤ 8 | 16 | ≥32 |  |  |
|  | Gentamicin | ≤ 4 | 8 | ≥16 |  |  |
|  | Cefepime | ≤2 | - | ≥ 16 |  |  |
|  | Amikacin | ≤16 | 32 | ≥64 |  |  |
|  | Amoxicillin+clavulanic acid | ≤8/4 | 16/8 | ≥32/16 |  |  |
|  | Cefuroxime | ≤ 8 | 16 | ≥32 |  |  |
|  | Cefotaxime | ≤1 | 2 | ≥4 |  |  |
|  | Ceftriaxone | ≤1 | 2 | ≥4 |  |  |
|  | Cefoxitin | ≤8 | 16 | ≥32 |  |  |
|  | Ceftazidime | ≤4 | 8 | ≥16 |  |  |
|  | Imipenem | ≤1 | 2 | ≥4 |  |  |
|  | Ertapenem | ≤0.5 | 1 | ≥2 |  |  |
|  | Meropenem | ≤1 | 2 | ≥4 |  |  |
|  | Ciprofloxacin | ≤1 | 2 | ≥4 |  |  |
|  | Trimethoprim+Sulfamethoxazole | ≤2/38 | - | ≥4/76 |  |  |
|  | Pipracillin-Tazobactam | ≤16/4 | 32/4–64/4 | ≥128/4 |  |  |
|  | Aztreonam | ≤4 | 8 | ≥16 |  |  |

1. Screening test for ESBL and AmpC producing gram negative bacteria on phoenix system:

A. Potential ESBL B. Potential AmpC β-lactamase producer

9. ESBL confirmation using combination disk test

A. POS B. NEG

10. AmpC confirmation using disc diffusion method

A. POS B. NEG
